# Proposing adjustments to heat safety thresholds for junior high and high school sports clubs in Japan

**DOI:** 10.1007/s00484-024-02812-4

**Published:** 2024-10-28

**Authors:** Takahiro Oyama, Yasushi Honda, Minoru Fujii, Kenichi Nakajima, Yasuaki Hijioka

**Affiliations:** 1 Center for Climate Change Adaptation, National Institute for Environmental Studies, 16-2, Onogawa, Tsukuba, 305-8506 Japan; 2 Social Systems Division, National Institute for Environmental Studies, 16-2, Onogawa, Tsukuba, 305-8506 Japan; 3 Material Cycles Division, National Institute for Environmental Studies, 16-2, Onogawa, Tsukuba, 305-8506 Japan

**Keywords:** Heat illness, Sports club activities, Wet-bulb globe temperature, Case-crossover study, Heat safety thresholds

## Abstract

With higher temperatures expected in the future due to global climate change, addressing health risks such as heat illness is increasingly important. In Japan, thousands of heat illness cases occur annually during school sports club activities. The risk may vary by sport, location, and region, but how heat safety thresholds (HSTs) should be adjusted to provide effective guidelines remains uncertain. Thus, we conducted a case-crossover study using data of heat illness cases and wet-bulb globe temperature (WBGT) throughout Japan to evaluate the heat illness risk for the current HSTs and propose adjustments. A significant relationship was found between heat illness incidence and WBGT at the time of the incident, as well as the average WBGT one and two days prior. The risk significantly varies with factors such as club, region, location, year, month, and the average WBGT in summer. Therefore, we recommend lowering the current HSTs by one category (3 °C) in the following cases: (1) clubs at high risk (baseball, softball, soccer/futsal, tennis, track and field, kyudo, and other with sustained exercise or thick uniforms); (2) from April to June; (3) in cooler regions (Hokkaido, Tohoku, Hokuriku, or where the average WBGT in summer≦18℃); (4) for outdoor activities; (5) when heat rapidly increases without adequate heat acclimatization. These findings may inform educators, students, sports authorities, and policymakers in adjusting HSTs to reduce the incidence of heat illness, thereby ensuring safer environments for school sports activities.

**Supplementary Information:**

The online version contains supplementary material available at 10.1007/s00484-024-02812-4.

## Introduction

For adolescents, physical activity has both short-term benefits, such as improved skeletal and mental health, and long-term benefits, such as the promotion of healthy lifestyles and reducing chronic diseases in adulthood (Hallal et al. 2006). However, during exercise, metabolism increases, and more than 80% of the energy is converted into heat (Hargreaves and Spriet 2020). This increases the risk of exertional heat stroke (EHS), the most serious form of exertional heat illness (EHI), due to both extrinsic (environment, clothing type, pressure from coaches, etc.) and intrinsic factors (heat acclimatization, fitness, hydration, etc.) (Adams 2019). Considering the predicted increase in the frequency and intensity of extreme heat due to global climate change (IPCC 2021), strategies to mitigate health risks, such as EHI, during exercise are becoming increasingly important. In Japan, 74.0% (boys) / 49.8% (girls) of junior high school students and 53.8% (boys) / 33.5% (girls) of high school students participate in school sports clubs (Sasakawa Sports Foundation 2022), and thousands of heat illness cases occur annually.

Despite the nationwide standard for exercise decision-making based on the wet-bulb globe temperature (WBGT) (Japan Sport Association 2019), adjusting heat safety thresholds (HSTs) that consider conditions beyond environmental factors (such as sport type, location, region, and school) remains a challenge (Casa et al. 2015). Although many sports federations have developed WBGT-based activity modification guidelines to reduce the risk of EHI (Racinais et al. 2015), these are general recommendations and not necessarily based on scientific evidence. Some validation and revision proposals of HSTs have been conducted for specific sports and conditions. The following are examples of the HSTs validation and revision in specific sports events or activities: Marathons held at latitudes above 40°N, where participants are not acclimatized to the heat, often result in race cancellations and many non-finishers when the WBGT exceeds 21 °C at the start (Roberts 2010); The incidence of significant heat illness among athletes competing on the FIVB Beach Volleyball World Tour was remarkably low, despite the WBGT often exceeding 32 °C, suggesting that the current HST of WBGT 31 °C may be too conservative in this case (Bahr and Reeser 2012); A high incidence rate was observed in American football at universities in the southeastern United States when the WBGT was 23–28 °C or above 28 °C, suggesting an influence of protective equipment and player size, and the effects of lack of heat acclimatization (Cooper et al. 2006); An increased risk of EHI was found in high school American football in north central Florida early in the season (presumably due to inadequate heat acclimatization) and when the WBGT exceeded 27.8 °C (82°F) (Tripp et al. 2021). There are also several quantitative methods proposed for the HSTs validation. Based on the simulation of core body temperature during sports using a thermo-physiological model, lowering the HSTs was proposed for marathon, triathlon, and soccer, by a few degrees for healthy general sports competitors in their 20 ~ 30 s (Oyama et al. 2023). To verify HSTs across different sports, methods based on the 90th percentile WBGT during the warm season in regions of the continental USA (Grundstein et al. 2015) and those based on the energy balance of the human body (Cheng et al. 2020) have been proposed. Still, it is questionable whether these findings can be directly applied to sports club activities in Japanese junior and senior high school, where a variety of sports are played and regional characteristics differ from those focused on in previous studies. Additionally, the analysis of the relationship between weather factors and heat illness risk in Japan faces selection bias issues, particularly when using newspaper-reported cases (Nakai et al. 2007). This concern extends to population-based studies in major Japanese cities (Iwashita 2018), where ecological fallacies may arise.

In this study, we conducted a case-crossover study using case data from 2010–2019 of the Japan Sport Council's injury and accident mutual aid benefit system (JSC-System), which covers approximately 95% of children and students in Japan, and hourly WBGT data from more than 800 sites since 2010 on the website of the Ministry of the Environment in Japan (MOEJ). Our objective was to evaluate the heat illness risk for the current HSTs during sports club activities in Japanese junior high and high schools and to propose adjustments to HSTs according to the conditions of the activity.

## Materials and methods

### Data selection

#### Heat illness incident data

We obtained data on heat illness incidents from 2010 to 2020 from the JSC-System. This system records school incidents requiring mutual aid benefits, covering junior high and high schools across Japan. Therefore, the study area was set as the entire country of Japan. The data are aggregated by municipality, and personally identifiable information such as school names or accident details are excluded. To reduce errors due to differences in WBGT data and the actual site conditions, only data where the distance between each municipality's representative location and the WBGT data site, which is described later, is within 10 km was used. The items in the data are explained in Table 1. We extracted data for incidents that (1) occurred during sports club activities, (2) were labeled as “heat illness” or “heat stroke” (in Japanese) based on medical doctors’ diagnosis, and (3) the mutual aid was provided between 2011 and 2019. According to the Japanese Association of Acute Medicine Heat-Related Illness criteria, heat illness is defined as "a general term for conditions caused by a disturbance in the body's adaptation to a hot environment," and heat stroke is the most severe of these conditions (Hifumi et al. 2018). These criteria were set to analyze the data by type of club, to focus on heat illness cases, and to avoid confounding factors related to the COVID-19 pandemic. Out of 47,084 total cases, 20,216 met these criteria.

**Table 1:** The contents of the heat illness incident data in Japanese junior high and high schools (2010–2019) (incident data provided by the Japan Sport Council)

Item	Contents
Fiscal aid year	Fiscal year in which mutual aid was provided
Prefecture name	Name of the prefecture where the incident occurred
Municipality name	Name of the municipality where the incident occurred
Date	Date the incident occurred
Time	Time the incident occurred
Type of school	Type of school where the incident occurred (including "junior high school," "full-time high school," "correspondence high school," and "part-time high school." In this study, the data for "correspondence high school" and "part-time high school" were minimal; therefore, these school types were combined with "full-time high school" under "high school.")
Location	Location where the incident occurred (20 types, including "sports field/schoolyard," "gymnasium/indoor sports facility," "off-school sports field/stadium." In this study, categories with fewer than 100 incidents were combined under "other.")
Region^*^	The region where the incident occurred (here we adopt the twelve regions in Japan based on climatic conditions, according to the Japan Meteorological Agency’s general information on the climate of Japan (https://www.data.jma.go.jp/cpd/longfcst/en/tourist.html, accessed May 15, 2024))
Club	Type of club activity during which the incident occurred (19 types, including "track and field club," "tennis club (including soft tennis)," "baseball club (including softball)," "soccer/futsal club," "basketball club." In this study, categories with fewer than 100 incidents were combined under "other.")
Circumstances	Circumstances in which the incident occurred (data from "sports club activities" were extracted)
Name of injury or disease	Type of injury or disease diagnosed by medical doctors (in this study, data containing the terms "heat illness" or "heat stroke" were extracted)

^*^: Regions were categorized by the authors based on prefecture information

#### Wet-bulb globe temperature data

**Fig. 1:**
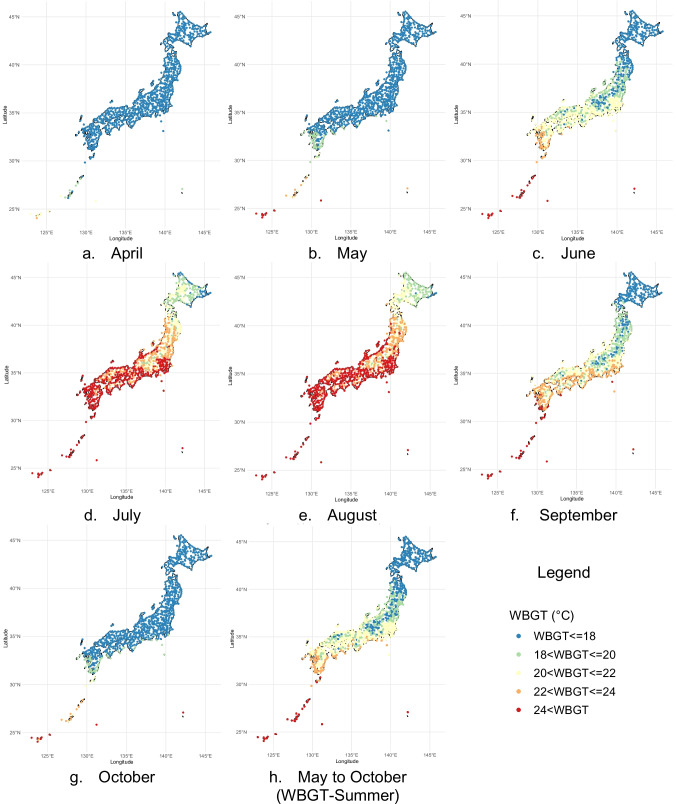
Monthly average wet-bulb globe temperature (WBGT) category from April to October 2010 ~ 2021 and the average WBGT from May to October 2010 ~ 2021 (WBGT-Summer) of each site in Japan where WBGT is estimated by Ministry of the Environment, Japan

We utilized WBGT, which is a common composite heat index in Japan for exercise decision-making (Yaglou and Minard 1957). WBGT data from 2010 to 2021 were obtained from the MOEJ's heat illness prevention information website (https://www.wbgt.env.go.jp/), offering data from more than 800 sites nationwide. Except for eleven sites that provide measured WBGT data, other sites provide only estimated WBGT data, so we used estimated data for all sites to ensure consistency. These estimates are calculated using Ono and Tonouchi's Eq. 1 (Ono and Tonouchi 2014), based on air temperature, relative humidity, solar radiation, and wind speed. The results of validation using three years of WBGT data for six cities from various regions showed that the correlation between the estimated value using Eq. 1 and the observed value was very high (R^2^ = 0.998), and 97.6% of the estimation errors were within ± 1 °C. Therefore, it was concluded that this equation is widely applicable for estimating summer WBGT throughout Japan (Ono and Tonouchi 2014). The location of the WBGT sites was obtained from the corresponding observation points in the Automated Meteorological Data Acquisition System (AMeDAS) of the Japan Meteorological Agency (Japan Meteorological Agency 2024). Figure 1 shows the WBGT category for each month from April to October 2010 ~ 2021 and the average WBGT from May to October 2010 ~ 2021 (WBGT-Summer) plotted on the location of WBGT sites throughout Japan. WBGT tends to be highest nationwide through July and August, while WBGT tends to be low in high latitude and mountainous regions.

Equation 1 Formula for estimating the wet-bulb globe temperature using generally measured meteorological indices (Ono and Tonouchi 2014)

1 $$WBGT=0.735{\times T}_{a}+0.0374\times RH+0.00292\times {T}_{a}\times RH+7.619\times SR-4.557\times {SR}^{2}-0.0572\times WS-4.064$$

Abbreviations: *RH* relative humidity (%), *SR* solar radiation (kW/m^2^), *T*_*a*_ air temperature (°C), *WBGT* wet-bulb globe temperature (°C), *WS* wind speed (m/s)

### Case-crossover analysis without stratification

In this study, traditional case‒control and cohort study methods could not be used due to the absence of control or follow-up data. Therefore, we conducted a case-crossover study (Maclure 1991), which is a self-controlled study design that allows the generation of control data from case data (Cadarette et al. 2021) and is also suitable for the analysis of acute illness associated with short-term exposure (Lewer et al. 2022) such as heat illness, with observation windows based on the time of onset. This approach allows for analysis using only case data and eliminates confounding factors such as fixed characteristics (e.g., gender, race) and those that change over time (e.g., height, age). This method necessitates a balanced time interval between case and control periods that is short enough to ensure interchangeability yet long enough to avoid short-term autocorrelation and intraindividual carryover effects (Mostofsky et al. 2018). Considering the short-term cumulative effect of heat on heat illness risk (Wallace et al. 2005) and the potential variability in school club activities by day of the week (DOW), we matched each heat illness case with up to four control periods by year, month, DOW, and hour, generating a dataset of 69,527 entries from 15,459 cases without missing values. We assumed that there were no heat illness incidents during the control periods due to the rarity of the illness.

We conducted statistical analysis using conditional logistic regression, which is suitable for predicting the binary outcome of heat illness incidents and handling multiple explanatory variables. The analyses were programmed using R version 4.2.3, with a significance level of *p* < 0.05. Twelve candidates for explanatory variables included the nearest hour WBGT category at the time of the heat illness incident (WBGT-0, one to five according to the categories "Guidebook for Prevention of Heat Illness during Sports Activities (JSA-Guideline)" of the Japan Sport Association [6]) and the categorized average WBGTs of the previous day (WBGT-1) and two days prior to the incident (WBGT-2), along with the type of school, club, region, location, month, year, DOW, hour, and WBGT-Summer (see Fig. 1 and Supplementary Fig. 1 for its distribution throughout Japan.) (Wallace et al. 2005; Nakai et al. 2007; Iwashita 2018). WBGT-1 and WBGT-2 were categorized in 1 °C increments with a lower limit of less than 15 °C because of the potentially complex effects on EHI risk on the day of the incident (e.g., if a high WBGT occurred the days before, the exercise content on the incident day may be adjusted). The Group Lasso method (Yuan and Lin 2006) was employed to identify the most predictive variables. Odds ratios were calculated based on the JSA-Guideline of the Japan Sport Association (Japan Sport Association 2019), using WBGT < 21 °C (the "almost safe" category in the JSA-Guideline) as a reference point. The 95% confidence intervals (95%CIs) for the odds ratios were calculated using Wald's method.

### Case-crossover analysis with stratification

The JSA-Guideline (Japan Sport Association 2019) recommends ceasing any exercise when WBGT is higher than 31 °C (31℃ < WBGT) and ceasing intense exercise when WBGT is higher than 28 °C but equals to or below 31 °C (28 < WBGT≦31 °C). These current HSTs are intended to be average guidelines and they lack explicit operational recommendations to consider specific conditions of the club activities. To address this, we conducted a stratified analysis by dividing the data into subsets (strata) based on the same explanatory variables as the analysis without stratification. For each combination of two strata, we assessed whether one of the odds ratios was significantly higher than the other by the Wald test, which uses the mean and standard error of the logarithmized odds ratios. To account for multiple testing, we corrected for type I errors using both the conservative Bonferroni method (Haynes 2013) and the less conservative Benjamini–Hochberg method (Benjamini and Hochberg 1995). We assessed significant differences in the odds ratios between strata at HSTs of “28 < WBGT≦31 °C” and “31 °C < WBGT”. For strata with significantly higher odds ratios, indicating higher risk of heat illness, we determined that applying the current HSTs was not appropriate. We proposed to lower the HSTs in the high-risk stratum with minimal changes, sufficient to eliminate significant differences by the Wald test noted above. Other methods and data used were the same as those used in the case-crossover study without stratification.

## Results

### Case-crossover analysis without stratification

**Fig. 2:**
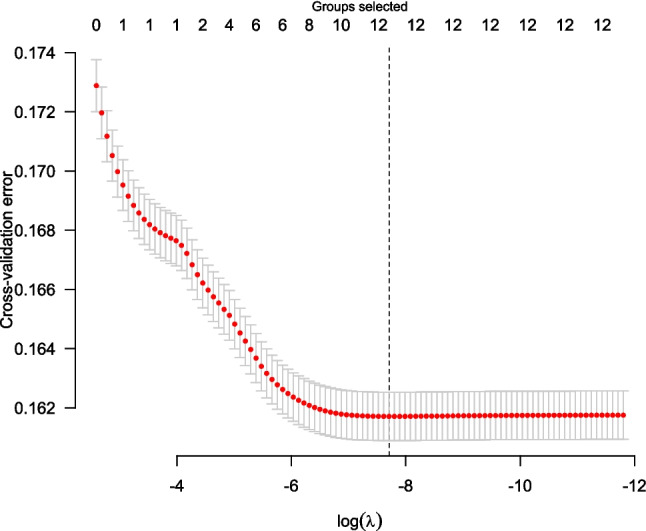
Cross-validation errors against log (λ) for the regularization parameters when the Group Lasso method was applied to the twelve candidate explanatory variables

We applied the Group Lasso method (Mostofsky et al. 2018) to the twelve candidate explanatory variables, namely, the WBGT-0, WBGT-1, WBGT-2, type of school, club, region, location, year, month, DOW, hour, and WBGT-Summer. All the twelve variables were selected for their contribution to the prediction accuracy. Figure 2 illustrates the cross-validation errors for the log-transformed regularization parameter log(λ), indicating the smallest error with these twelve explanatory variables.

We conducted a conditional logistic regression analysis using the twelve selected explanatory variables to predict heat illness occurrence. Cases were matched with up to four controls according to year, month, DOW, and hour. The model showed a good predictive accuracy with a concordance of 0.682 (standard error (SE) = 0.003). The model also fit well, as indicated by the likelihood ratio value of 5,370, the Wald test value of 4,303, and the score (log-rank) test value of 4,901, all with 33 degrees of freedom (df), *p* < 2e-16. Only small multicollinearity was noted for WBGT-0 (variance inflation factor = 1.55). Among the variables, the WBGT-0, WBGT-1, and WBGT-2 were significant at *p* < 0.05 (Table 2). The positive coefficients for two of these variables (WBGT-0 and WBGT-1) indicated that the increases in these indices associated with a greater probability of heat illness. For example, for each 1 °C increase in the WBGT-0, the probability of heat illness increased by approximately 103%. Similarly, the probability of heat illness increased with higher WBGT-1. A WBGT-1 of 28 °C was associated with a 644% increase in the probability of heat illness compared to a WBGT-1 of less than 15 °C (the reference category), for example. On the other hand, the coefficients for many of the WBGT-2 categories were negative and significant, indicating that higher temperatures two days prior were associated with a decreased probability of heat illness. For example, compared to a WBGT-2 of less than 15 °C, a WBGT-2 of 28 °C was associated with a 35% decrease in the probability of heat illness.

**Table 2:** Regression coefficients and log-transformed regression coefficients with 95% confidence intervals, standard errors, z values, and p values for significant explanatory variables: The significance level was set to *p* = 0.05

Explanatory variables	Coef.	Exp (Coef.) (95%CI)	SE (Coef.)	z value	*p* value
WBGT-0	0.71	2.03 (1.97–2.10)	0.02	42.48	< 2e-16^***^
WBGT-1 (15℃)	0.36	1.44 (1.12–1.85)	0.13	2.81	4.92e-3^**^
WBGT-1 (16℃)	0.48	1.61 (1.25–2.09)	0.13	3.62	2.90e-4^***^
WBGT-1 (17℃)	0.53	1.69 (1.29–2.22)	0.14	3.84	1.21e-4^***^
WBGT-1 (18℃)	0.59	1.81 (1.37–2.39)	0.14	4.15	3.35e-5^***^
WBGT-1 (19℃)	0.80	2.23 (1.68–2.97)	0.15	5.52	3.40e-8^***^
WBGT-1 (20℃)	0.85	2.34 (1.75–3.13)	0.15	5.71	1.15e-8^***^
WBGT-1 (21℃)	0.95	2.57 (1.91–3.47)	0.15	6.23	4.75e-10^***^
WBGT-1 (22℃)	0.90	2.47 (1.82–3.35)	0.16	5.82	5.90e-9^***^
WBGT-1 (23℃)	1.18	3.24 (2.38–4.41)	0.16	7.47	7.84e-14^***^
WBGT-1 (24℃)	1.29	3.65 (2.67–4.99)	0.16	8.10	5.43e-16^***^
WBGT-1 (25℃)	1.48	4.37 (3.19–6.00)	0.16	9.14	< 2e-16^***^
WBGT-1 (26℃)	1.65	5.23 (3.79–7.20)	0.16	10.13	< 2e-16^***^
WBGT-1 (27℃)	1.82	6.19 (4.47–8.57)	0.17	11.00	< 2e-16^***^
WBGT-1 (28℃)	2.01	7.44 (5.32–10.40)	0.17	11.74	< 2e-16^***^
WBGT-1 (29℃)	1.90	6.68 (4.52–9.88)	0.20	9.51	< 2e-16^***^
WBGT-1 (30℃)	1.75	5.77 (1.37–24.26)	0.73	2.39	1.67e-2^*^
WBGT-2 (20℃)	-0.32	0.73 (0.55–0.97)	0.15	-2.15	3.14e-2^*^
WBGT-2 (21℃)	-0.33	0.72 (0.54–0.96)	0.15	-2.21	2.73e-2^*^
WBGT-2 (22℃)	-0.36	0.70 (0.52–0.94)	0.15	-2.40	1.63e-2^*^
WBGT-2 (24℃)	-0.41	0.66 (0.49–0.89)	0.15	-2.69	7.22e-3^**^
WBGT-2 (25℃)	-0.42	0.66 (0.49–0.89)	0.15	-2.71	6.64e-3^**^
WBGT-2 (26℃)	-0.41	0.67 (0.49–0.90)	0.16	-2.61	9.05e-3^**^
WBGT-2 (27℃)	-0.41	0.66 (0.49–0.90)	0.16	-2.62	8.73e-3^**^
WBGT-2 (28℃)	-0.43	0.65 (0.47–0.89)	0.16	-2.66	7.72e-3^**^
WBGT-2 (29℃)	-0.52	0.59 (0.41–0.87)	0.19	-2.70	6.90e-3^**^

*CI* confidence interval, *Coef.* regression coefficient, *Exp* exponential, *SE* standard error, *WBGT* wet-bulb globe temperature (℃)

*p*-value guide: ^***^ *p*≦0.001, ^**^ 0.001 < *p*≦0.01, ^*^ 0.01 < *p*≦0.05

**Fig. 3:**
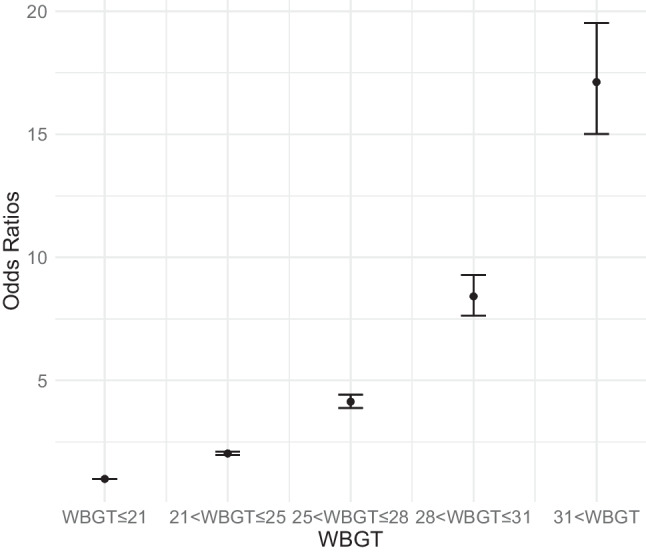
Odds ratios and 95% confidence intervals for heat illness incidents in five wet-bulb globe temperature categories (odds ratio of 1 for WBGT≦21 °C)

Figure 3 shows the odds ratios and 95%CIs for heat illness incidents at five WBGT categories based on the developed model. A clear trend was observed where higher WBGT categories corresponded to increased odds ratios. Using a category of WBGT < 21 °C as the reference (odds ratio of 1), the odds ratios were 8.42 (7.63–9.29) for 28 < WBGT≦31 °C and 17.12 (15.02–19.52) for 31 °C < WBGT.

### Case-crossover analysis with stratification

We formed groups with multiple strata based on nine variables (type of school, club, region, location, year, month, DOW, hour, and WBGT-Summer) among those selected by the Group Lasso method. For each stratified group, we calculated odds ratios within each combination of strata and assessed significant differences between them. Most strata in all groups exhibited a significant trend toward increasing odds ratios with higher WBGT-0, consistent with the pre-stratification results (Supplementary Figs. 2 through 10). Within the six groups stratified by variables such as club, region, location, year, month, and WBGT-Summer, there were significant differences among strata. The odds ratios at the categories of 28 < WBGT≦31 °C and 31 °C < WBGT for strata where lowering the HSTs were proposed, along with their 95%CIs, are presented in Tables 3 and 4. For all cases where there was a significant difference between strata, lowering the HSTs by one category (3 °C) means that the Bonferroni's method no longer detects a significant difference, so we proposed lowering the HSTs by one category only for such cases. Other results, including strata where no revision of the HSTs was proposed and the p-values after lowering the HSTs, are presented in Supplementary Table 1 and Supplementary Table 2. Because the Benjamini–Hochberg method is based on the rank of the p-value in order to control the false discovery rate, it is difficult to apply it dynamically by considering the p-value when lowering the HSTs of each stratum. Therefore, we did not use the B&H method to detect significant differences after lowering the HSTs. The group-specific results are summarized below:

- Club: Significant differences were observed in clubs of baseball, football/futsal, kyudo (Japanese archery), softball, tennis, and track and field activities compared to other clubs, respectively. To eliminate the significant differences, the WBGT category of 28 < WBGT≦31 °C should be adjusted to 25 < WBGT≦28 °C, and that of 31 °C < WBGT should be adjusted to 28 < WBGT≦31 °C for these clubs. Similar adjustments were made in other strata and groups, as described below.
- Region: Significant differences were observed in Hokkaido, Tohoku, and Hokuriku compared to other regions, respectively. To eliminate the significant difference, the WBGT category of 28 < WBGT≦31 °C should be adjusted to 25 < WBGT≦28 °C, and that of 31 °C < WBGT should be adjusted to 28 < WBGT≦31 °C for these identified regions.
- Location: Significant differences were observed in school playgrounds and schoolyards, out-of-school playgrounds and stadiums, and roads compared to other locations, respectively. To eliminate the significant difference, the WBGT category of 28 < WBGT≦31 °C should be adjusted to 25 < WBGT≦28 °C, and that of 31 °C < WBGT should be adjusted to 28 < WBGT≦31 °C for these identified locations.
- Year: For 2019, a significant difference was observed compared to 2017. To eliminate the significant difference, the WBGT category of 28 < WBGT≦31 °C should be adjusted to 25 < WBGT≦28 °C, and that of 31 °C < WBGT should be adjusted to 28 < WBGT≦31 °C for 2019.
- Month: Significant differences were observed in April–May and June compared to July and/or August. To eliminate the significant difference, the WBGT category of 28 < WBGT≦31 °C should be adjusted to 25 < WBGT≦28 °C, and that of 31 °C < WBGT should be adjusted to 28 < WBGT≦31 °C for these identified months.
- WBGT-Summer: Significant differences were observed in sites with WBGT-Summer of WBGT≦18℃ compared to other categories. To eliminate the significant difference, the WBGT category of 28 < WBGT≦31 °C should be adjusted to 25 < WBGT≦28 °C, and that of 31 °C < WBGT should be adjusted to 28 < WBGT≦31 °C for these relatively cool sites. See Fig. 1 for the distribution of sites by WBGT-Summer category.
- DOW, Hour, School: No strata were significantly different within these groups.

**Table 3:** Odds ratios with 95% confidence intervals and variables with significant differences, in strata where revisions were proposed to lower the HSTs by one category (for the current category of 28 < WBGT≦31℃ for ceasing strenuous exercise, with the odds ratio for WBGT≦21 °C set as 1)

EV1 (group)	EV2 (stratification)	OR (95%CI)	EV2 significantly different (p for Bonf.) ^a^	EV2 significantly different (p for B&H) ^b^	n
Club	Baseball	12.05 (9.61–15.11)	Basketball (4.22e-5)^*^, Volleyball (9.80e-7)^***^	Basketball (9.57e-4)^**^, Table tennis (1.96e-2)^*^, Volleyball (6.67e-5)^***^	14,110
	Football/Futsal	8.94 (6.82–11.71)	N/A	Volleyball (9.25e-3)^*^	9,203
	Kyudo (Japanese archery)	84.90 (22.28–323.50)	Basketball (1.04e-4)^*^, Table tennis (8.21e-5)^*^, Volleyball (2.50e-5)^*^	Badminton (8.27e-3)^*^, Baseball (2.51e-2)^*^, Basketball (1.76e-3)^**^, Football (9.25e-3)^*^, Handball (9.88e-3)^*^, Kendo (3.53e-3)^**^, Rugby (2.62e-2)^*^, Swimming (4.08e-3)^**^, Table tennis (1.59e-3)^**^, Tennis (1.97e-2)^*^, T&F (1.79e-2)^*^, Volleyball (8.50e-4)^**^, Other (5.83e-3)^*^	453
	Softball	22.34 (12.86–38.80)	Basketball (1.42e-5)^**^, Table tennis (1.45e-4)^*^, Volleyball (7.66e-7)^***^	Badminton (2.52e-2)^*^, Basketball (6.45e-4)^**^, Football (1.97e-2)^*^, Kendo (5.83e-3)^*^, Table tennis (2.19e-3)^**^, Volleyball (6.67e-5)^***^, Other (1.40e-2)^*^	2,775
	Tennis	11.05 (8.37–14.58)	Volleyball (3.18e-5)^*^	Basketball (8.27e-3)^*^, Table tennis (3.86e-2)^*^, Volleyball (8.66e-4)^**^	9,484
	T&F	10.36 (7.46–14.38)	N/A	Basketball (2.89e-2)^*^, Volleyball (3.98e-3)^**^	6,627
WBGT-Summer	WBGT≦18	19.39 (11.93–31.50)	20 < WBGT≦22 (6.38e-4)^*^, 22 < WBGT≦24 (4.68e-4)^*^	18 < WBGT≦20 (3.08e-2)^*^, 20 < WBGT≦22 (3.19e-3)^**^, 22 < WBGT≦24 (3.19e-3)^**^	3,241
Month	April–May	16.11 (10.01–25.94)	August (8.96e-4)^*^	July (3.63e-2)^*^, August (8.96e-3)^*^	3,860
	June	12.74 (8.94–18.17)	August (1.89e-3)^*^	August (9.46e-3)^*^	5,616
Region	Hokkaido	26.50 (11.86–59.22)	N/A	Kanto-Koshin (6.14e-3)^*^, Kinki (4.33e-3)^**^, Chugoku (4.36e-3)^**^, Shikoku (4.95e-3)^**^, Northern Kyushu (1.40e-3)^**^, Southern Kyushu and Amami (2.14e-3)^**^	1,283
	Tohoku	12.34 (8.64–17.63)	N/A	Northern Kyushu (3.75e-2)^*^	5,221
	Hokuriku	15.89 (10.19–24.76)	N/A	Kinki (4.16e-2)^*^, Northern Kyushu (3.75e-2)^*^, Southern Kyushu and Amami (3.75e-2)^*^	3,719
Location	Out-of-school playgrounds and stadiums	12.28 (10.17–14.82)	Gymnasium/Indoor sports ground (1.69e-7)^***^, Out-of-school sports halls (1.24e-7)^***^, Other (1.73e-6)^***^	Gymnasium/Indoor sports ground (1.77e-6)^***^, Out-of-school sports halls (1.77e-6)^***^, Other (1.21e-5)^***^	19,752
	Playgrounds and schoolyards	10.16 (8.60–12.00)	Gymnasium/Indoor sports ground (3.42e-5)^**^, Out-of-school sports halls (1.15e-5)^***^, Other (3.43e-5)^**^	Gymnasium/Indoor sports ground (1.20e-4)^***^, Out-of-school sports halls (6.05e-5)^***^, Other (1.20e-4)^***^	25,615
	Roads	13.97 (7.11–27.46)	Other (8.48e-4)^*^	Gymnasium/Indoor sports ground (2.99e-2)^*^, Out-of-school sports halls (9.75e-3)^*^, Other (2.54e-3)^**^	1,752
Year	2019	12.16 (8.61–17.18)	2017 (2.45e-4)^*^	2017 (1.10e-2)^*^	6,609

*Bonf.* Bonferroni’s method, *B&H* Benjamini-Hochberg’s method, *CI* confidence interval, *EV* explanatory variable, *OR* odds ratio, *WBGT* wet-bulb globe temperature (℃), α: significance levels

^a^: α of differences from other EV2s in the same EV1 (Bonf.): Club, 1.84e-4; Day of Week, 1.19e-3; Hour, 4.17e-3; Average WBGT from May to October, 2.50e-3; Month, 2.50e-3; Location, 1.19e-3; School, 2.50e-3; Year, 5.56e-4

^b^: α of differences from other EV2s in same EV1 (B&H): 0.05

p-value guide: ^***^ p≦α/100, ^**^ α/100 < p ≦ α/10, ^*^ α/10 < p≦α

**Table 4:** Odds ratios with 95% confidence intervals and variables with significant differences, in strata where revisions were proposed to lower the HSTs by one category (for the current category of 31℃ < WBGT for ceasing all exercise, with the odds ratio for WBGT≦21 °C set as 1)

EV 1 (group)	EV 2 (stratification)	OR (95%CI)
Club	Baseball	27.64 (20.44–37.37)
	Football/Futsal	18.55 (12.94–26.59)
	Kyudo	373.16 (62.70–2220.78)
	Softball	62.92 (30.14–131.34)
	Tennis	24.60 (16.99–35.63)
	Track and field	22.58 (14.59–34.96)
WBGT-Summer	WBGT≦18	52.09 (27.27–99.48)
Month	April–May	40.69 (21.56–76.79)
	June	29.77 (18.55–47.77)
Region	Hokkaido	79.00 (27.04–230.82)
	Tohoku	28.53 (17.74–45.89)
	Hokuriku	38.61 (21.48–69.39)
Location	Out-of-school playgrounds and stadiums	28.32 (22.04–36.39)
	Playgrounds and schoolyards	22.00 (17.62–27.47)
	Roads	33.64 (13.66–82.85)
Year	2019	27.97 (17.65–44.32)

*CI* confidence interval, *EV* explanatory variable, *OR* odds ratio, *WBGT* wet-bulb globe temperature (℃)

## Discussion

### Study findings

We conducted a case-crossover study using case data on heat illness incidents in junior high and high school sports club activities in Japan, along with WBGT data from nearby weather stations. The analysis of both pre- and post-stratification data confirmed a significant relationship between heat illness incidence and WBGT variables (WBGT-0, WBGT-1, and WBGT-2). This finding is consistent with previous research showing that many heat illness cases in Japan occur at a WBGT of 28 °C or higher (Nakai et al. 2007) and that the odds of heat illness in junior high and high school of major Japanese cities increase with higher WBGT (Iwashita 2018). Interestingly, the coefficients for many of WBGT-2 categories were negative and significant, indicating that higher temperatures two days prior were associated with a decreased probability of heat illness. This suggests that higher WBGTs two days prior to the incident might lead to behavioral changes that reduce the risk on the incident day. These results suggest that accuracy in analyzing the risk of heat illness in sports activities may be improved by considering not only the WBGT at the time of the event, but also the WBGT up to several days prior. By applying a case-crossover approach to case data from the JSC-System, which covers most of the child and student population (Japan Sport Council 2021), we were able to confirm the significant relationship between the WBGT variables and heat illness incidents during school sports club activities, mitigating the risks of selection bias and ecological fallacy associated with the previous literature (Nakai et al. 2007; Iwashita 2018).

In the stratified analysis, significant differences in odds ratios were observed within strata in groups stratified by club, region, location, year, month, and WBGT-Summer. This indicates that it is important to adjust the HSTs to account for the above variables in order to manage the risk of heat illness.

High odds ratios were observed for baseball, football/futsal, kyudo (Japanese archery), softball, tennis, and track and field clubs. It is interesting to note that the odds ratios were high not only for clubs named after sports that require continuous exercise and are played outdoors (football/futsal, tennis, and track and field). Although not directly supported by this study, the high odds ratios in baseball, softball, and kyudo may possibly be due to the continuous high metabolic demand during practice, which is a large part of club activities, and the relatively thick uniforms that cover large parts of the body and impede heat dissipation. Further attention should be paid to the results for archery, as the confidence intervals are very wide probably due to the limited sample size (*n* = 453). The higher odds ratios in the early months (April to June), in northern regions (Hokkaido, Tohoku, and Hokuriku), and in sites where WBGT-Summer equals to or is less than 18℃(Ministry of the Environment Japan n.d.) consistently suggest that heat illness risk in sports club activities increases when heat acclimatization is insufficient and the WBGT is high at the same time. This aligns with the finding that heat illness occurs in June at lower temperatures and humidity than in July (Nakai et al. 2007), and higher latitude areas in Japan have more heat illness cases than lower latitude areas at a maximum daily WBGT of 33 °C (Oka et al. 2023). The analysis also revealed that higher odds ratios were observed when club activities were conducted outdoor locations, suggesting an increased risk of heat illness with greater exposure to outdoor heat. All three outdoor categories, "Playgrounds and schoolyards," "Out-of-school playgrounds and stadiums," and "Roads," had significantly higher odds ratios than the indoor categories, "Gymnasium/Indoor sports ground," and "Out-of-school sports halls.” Although the installation rate of air conditioners in school gymnasiums is still low in Japan (about 10%) [28], the risk of heat illness may be reduced indoors compared with outdoors, where direct sunlight can be avoided, and environmental conditions can be controlled. For the year, 2019 had a higher average odds ratio than all other years and significantly higher than 2017. It is deduced that in 2019, the relatively cool month of July caused people to become insufficiently heat acclimatized and August became very hot, resulting in a large number of heat illness cases nationwide (Ministry of the Environment Japan n.d.) (See Supplementary Fig. 11 for monthly mean WBGT in Japan from 2010 to 2021). Such changes in the risk of heat illness due to annual changes in the heat environment are likely to be reflected in the results of this study. Although no significant differences in odds ratios were observed by day of the week, the odds ratio on Mondays were relatively small and may be influenced by the restorative physical fitness of the weekend.

These findings highlight the need for the flexible adjustments of HSTs. Specifically, the categories of 28 < WBGT≦31 °C for "ceasing strenuous exercise" and that of 31 °C < WBGT for "ceasing all exercise" should be adjusted considering factors such as club, region, location, year, month, and WBGT-Summer.

### Clinical implications

Every year, thousands of heat illness cases in Japanese junior high and high school sports clubs highlight the need for enhanced countermeasures. The odds ratios for heat illness obtained in this study, which increase rapidly as WBGT rises, reinforce the need for compliance with the current HSTs as a basis. In particular, if the WBGT is between 28 °C and 31 °C, strenuous exercise should be ceased, and if the WBGT is 31 °C or higher, all exercise should be ceased. Key measures include consistent WBGT monitoring and responsive actions at club activity. In a prefecture in Japan's Kanto region, approximately 90% of junior high schools measure WBGT, with 64.1% taking action based on the measurement (Kubo and Akaogi 2023). However, a broader nationwide understanding is still needed.

The stratified analysis in this study showed that the risk of heat illness varies according to factors such as clubs, regions, locations, months, WBGT-Summer, and years. This underscores the need for adjustments of HSTs in school sports clubs, adapting to specific contexts, in addition to the compliance with the existing HSTs. It is recommended to lower the HSTs by one category for; (1) clubs at high risk of heat illness (baseball, softball, soccer/futsal, tennis, track and field, kyudo, and other with sustained exercise or thick uniforms); (2) months from April to June; (3) cooler regions (Hokkaido, Tohoku, Hokuriku, or where WBGT-Summer≦18℃); (4) outdoor activities; (5) when heat rapidly increases while heat acclimatization is inadequate (e.g., a cool July followed by a hot August, as in 2019). Factors such as water deficiency, excessive physical exertion, lack of rest, inappropriate clothing, and protective equipment that hinders heat dissipation are risk factors for exertional heat illness in adolescents (Bergeron et al. 2011). Thus, along with lowering HSTs, establishment of a heat acclimatization period with short practice time and less protective equipment (Cooper et al. 2020), enhancing cooling measures to suppress body temperature increases before (e.g., cold water/ice slurry ingestion), during (e.g., facial wind/water spray, ice vest, menthol cooling), and after activities (e.g., cold water immersion) (Bongers et al. 2017), promoting indoor practices in air-conditioned environments, and implementing more frequent and shorter breaks are also important strategies. For example, in high school American football in the hot and humid state of Georgia, adherence to WBGT-based activity modification categories and a mandatory 5-day heat acclimatization period with short practice times reduced heat syncope and heat exhaustion illness rates by 35% to 100%, depending on the WBGT category (Cooper et al. 2020). Such combined heat illness measures could be effective in Japan as well. In addition, in order to spread the flexible adjustments of the HSTs, it is considered important to call through influential organizations such as the Japan Sport Association, which developed the current HSTs that are referenced by a wide range of organizations including Nippon Junior High School Physical Culture Association, All Japan High School Athletic Federation, and national federations for various sports.

### Limitations

This study analyzed heat illness (mainly EHI) incidents from 2010 to 2019 during junior high and high school sports club activities in Japan. Therefore, it has not been confirmed whether the observed relationship between the WBGT and heat illness is applicable to other years, age groups, countries, or classic heat illness. Still, the method of the study is applicable when data on heat illness incidents, including time and geographic information, and WBGT (or basic weather indices for estimation of WBGT (Liljegren et al. 2008; Ono and Tonouchi 2014)) data are available, and could contribute to the study of quantitative evaluation of heat illness risk and countermeasures under conditions other than those covered in this study. Future research should expand to a broader demographic and context, especially considering potential changes after the COVID-19 pandemic. Because children have a larger relative skin area than adults and may be more susceptible to thermal injury in extreme environments (Falk and Dotan 2008), children younger than those targeted in this study (e.g., elementary school children and infants) should also be prioritized for future research. The analyzed data lacked details on conditions during heat illness incidents, such as specific exercise, metabolic rates, cooling measures, rest periods, duration, or individual’s physical/mental conditions. Hence, the results may be influenced particularly by factors such as rigorous training, unusual activities (such as punishment runs), exercising in poor health, or effective cooling. Additionally, in stratified analysis, some strata had small sample sizes, which may have affected confidence intervals and statistical significance. For example, the odds ratio in the kyudo club was very high compared to the others, but the confidence interval was very wide probably due to the limitations of the sample size (*n* = 453), and there is room to research whether the result reflect reality. Through more comprehensive surveillance of heat illness incidents, it is expected that detailed circumstances can be explicitly taken into account, increasing the credibility of future studies on the relationship between heat illness and environmental conditions and, in turn, enabling more specific heat stroke prevention recommendations (Hosokawa et al. 2021). There might be an error between the WBGT based on the weather station data used in this study and the WBGT at the exact locations where the cases occurred. In addition, the matching of heat illness cases to WBGT data at the municipality level may not reflect the exact location and surrounding environment of the incident, thus affecting the results. As countermeasures to the possible WBGT error, we limited the analysis to cases where the distance from the station to the municipality was 10 km or less, and included the location categories (e.g., playground, gymnasium, park) in the explanatory variables of the model to indirectly account for solar radiation, wind speed, and surrounding land use (Hsu et al. 2023) that affect the local WBGT. More detailed information of the incidents such as latitude, longitude, land use, and elevation, ideally with local WBGT measurements, may provide a more accurate analysis. Finally, since WBGT may underestimate heat stress in environments where sweat evaporation is limited, such as high humidity and low wind speed (Budd 2008), future research could assess heat illness risk more accurately by considering humidity and wind speed directly in the analysis.

## Conclusions

Our case-crossover study, in which data on heat illness incidents in junior high and high school sports club activities across Japan and WBGT data from nearby locations were used, confirmed a significant relationship between heat illness incidents and WBGT at the time of the incident, as well as the average WBGT of the previous day, and two days before. The stratified analysis further suggested that the risk of heat illness varies by factors such as club, region, location, year, month, and WBGT-Summer. The novelty of this study is that it quantifies changes in the risk of heat illness in junior high and high school sports club activities across a variety of sports, considering various factors above, and also proposes specific countermeasures including the adjustments of the HSTs. To effectively manage the risk of heat illness tailored to factors of activities, it is recommended to lower the current HSTs by one category (3 °C) in case; (1) clubs at high risk of the heat illness (baseball, softball, soccer/futsal, tennis, track and field, kyudo, and other with sustained exercise or thick uniforms); (2) months from April to June; (3) cooler regions (Hokkaido, Tohoku, Hokuriku, or where WBGT-Summer≦18℃); (4) outdoor activities; (5) when heat rapidly increases while heat acclimatization is inadequate.

## Supplementary Information

Below is the link to the electronic supplementary material.

Supplementary file1 (DOCX 7531 KB)

## Data Availability

The WBGT data are available at https://www.wbgt.env.go.jp/wbgt_data.php. Other data generated and analyzed during the study are available from the corresponding author upon reasonable requests.
