# Supplementary material for: Proposing adjustments to heat safety thresholds for junior high and high school sports clubs in Japan
Source: Int J Biometeorol. 2024 Oct 28;69(2):343–55. doi: 10.1007/s00484-024-02812-4 (PMC11785657; doi:10.1007/s00484-024-02812-4)
Supplement: Supplementary file 1 — Supplementary file1 (DOCX 7531 KB) [file 484_2024_2812_MOESM1_ESM.docx]

**Supplementary information for “Proposing adjustments to heat safety thresholds for junior high and high school sports clubs in Japan”**

| 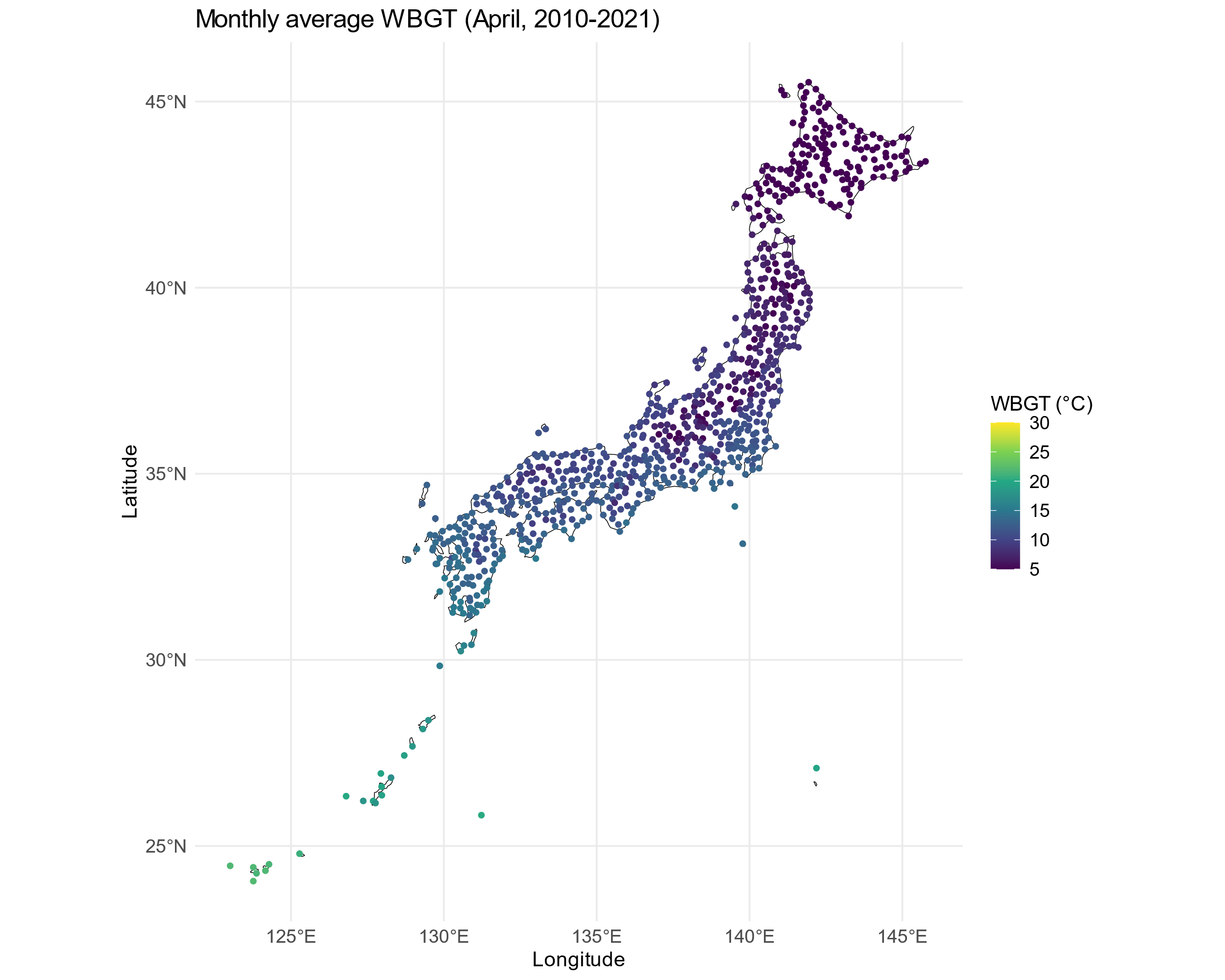 | 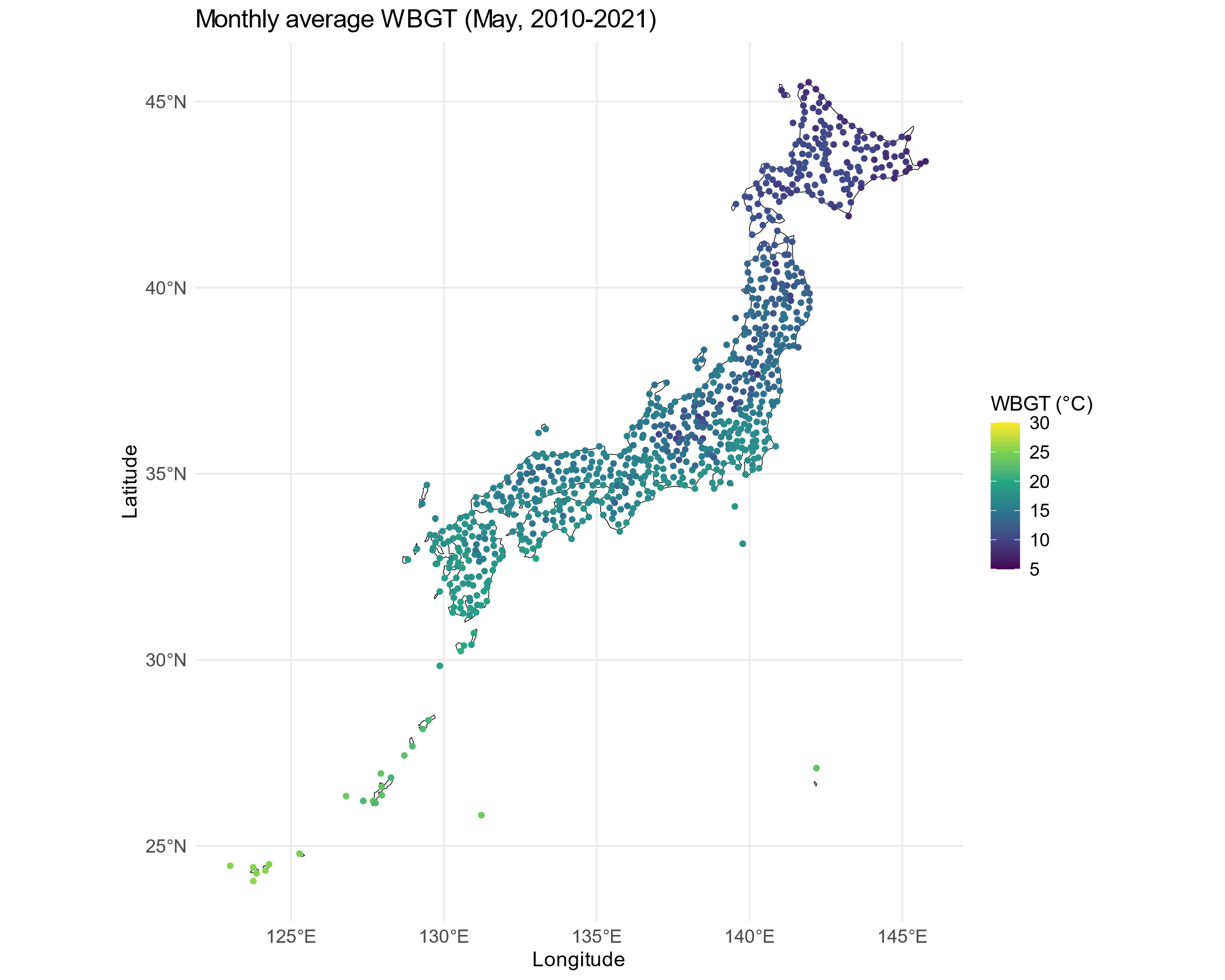 | 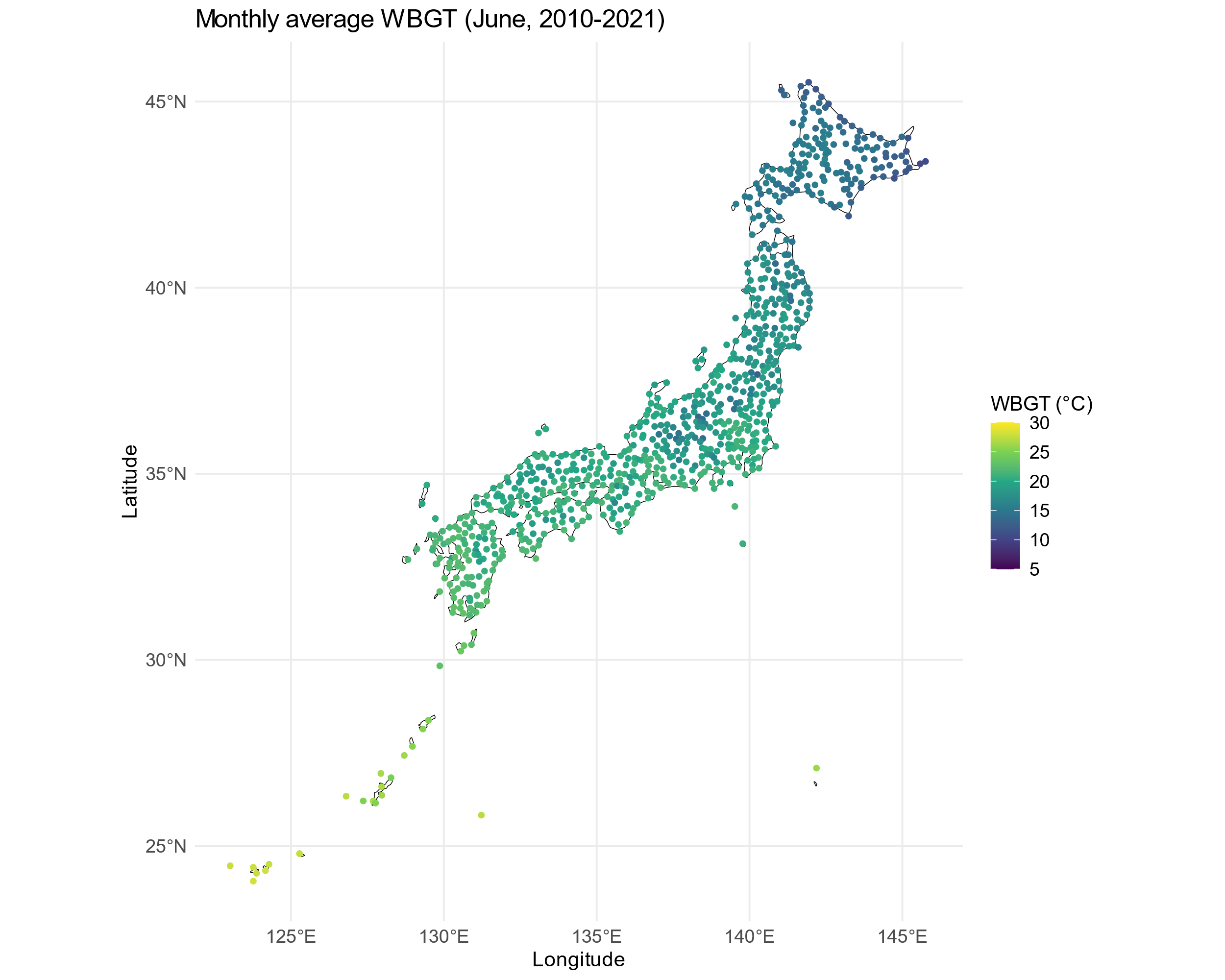 |
| --- | --- | --- |
| 1. April | 1. May | 1. June |
| 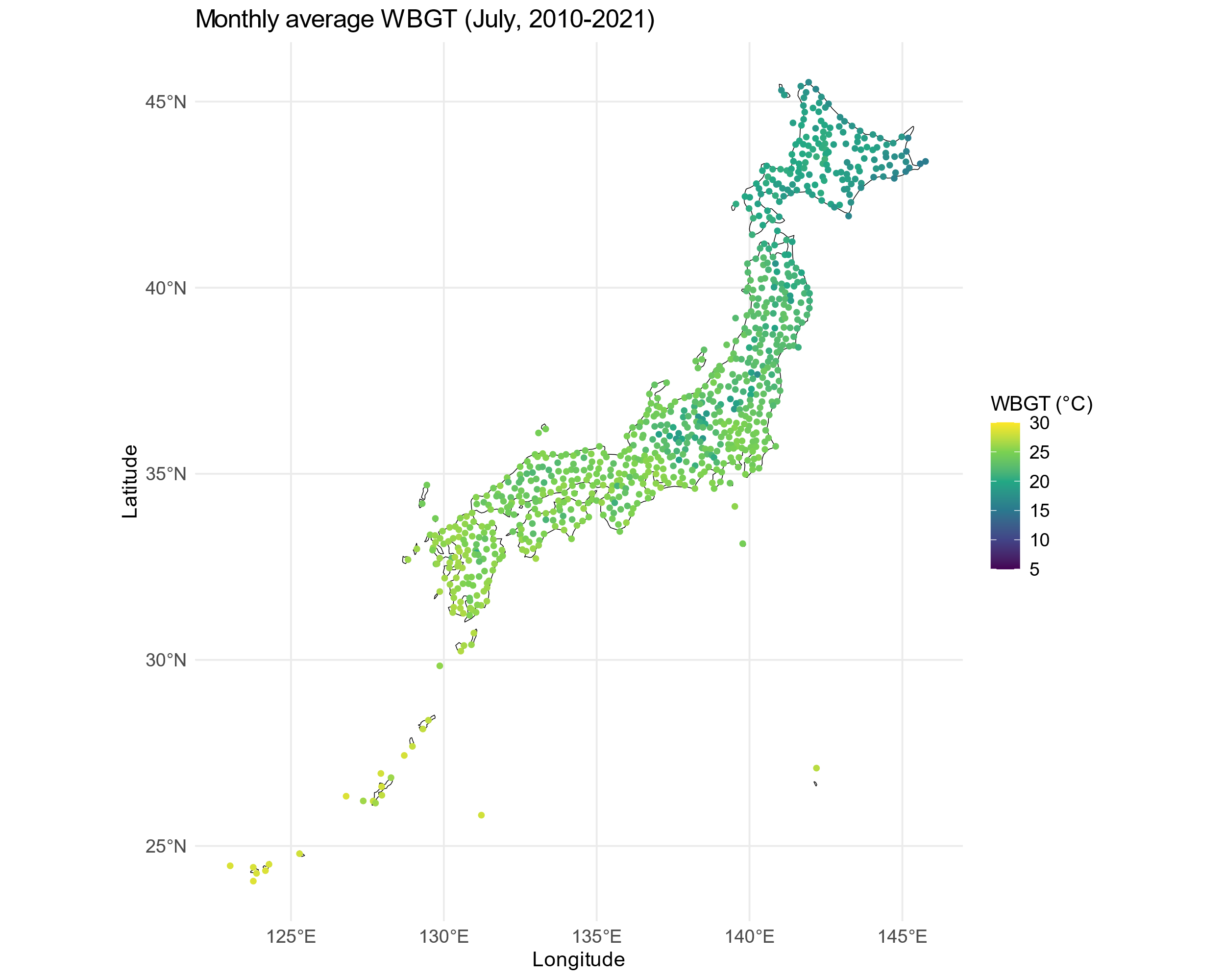 | 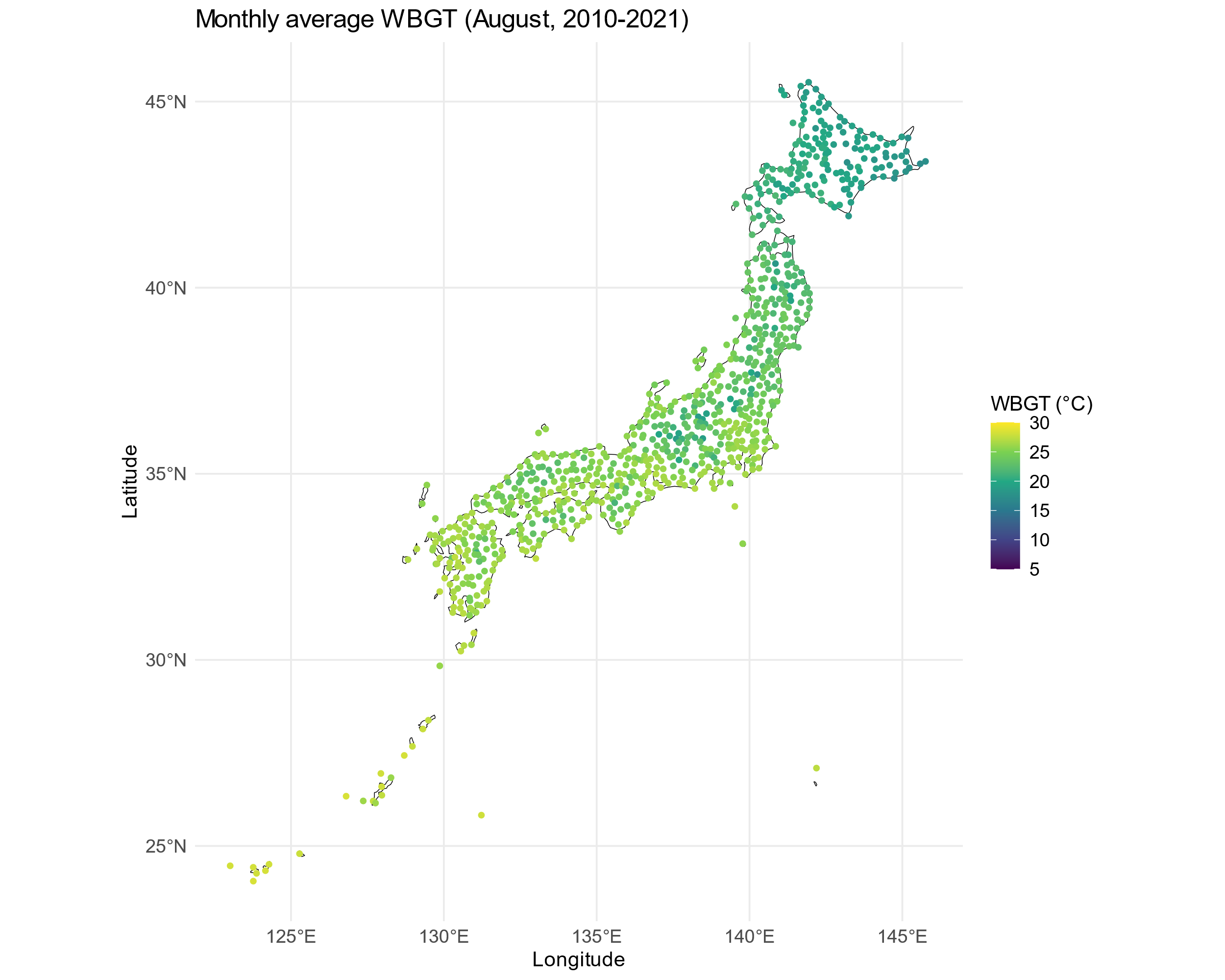 | 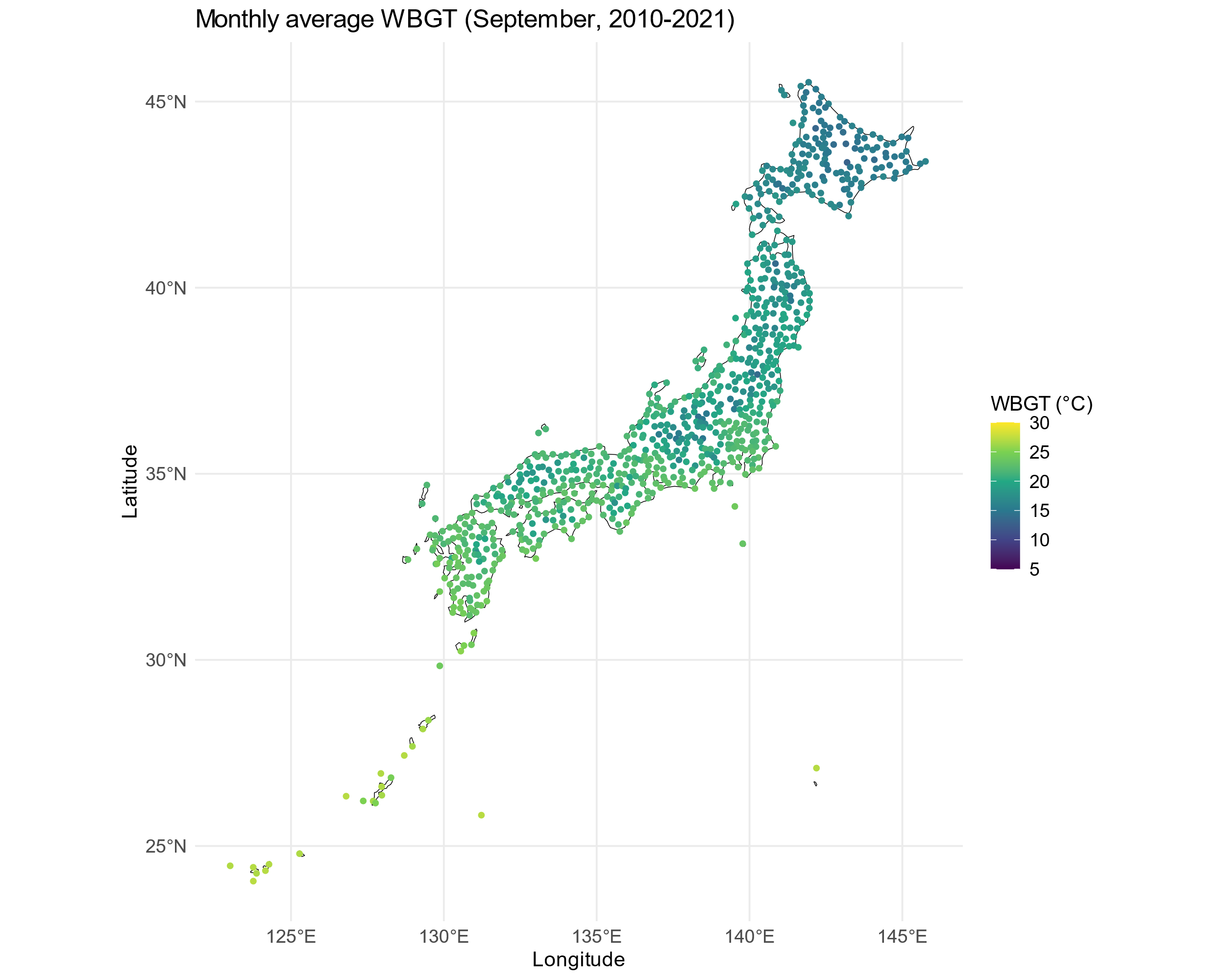 |
| 1. July | 1. August | 1. September |
| 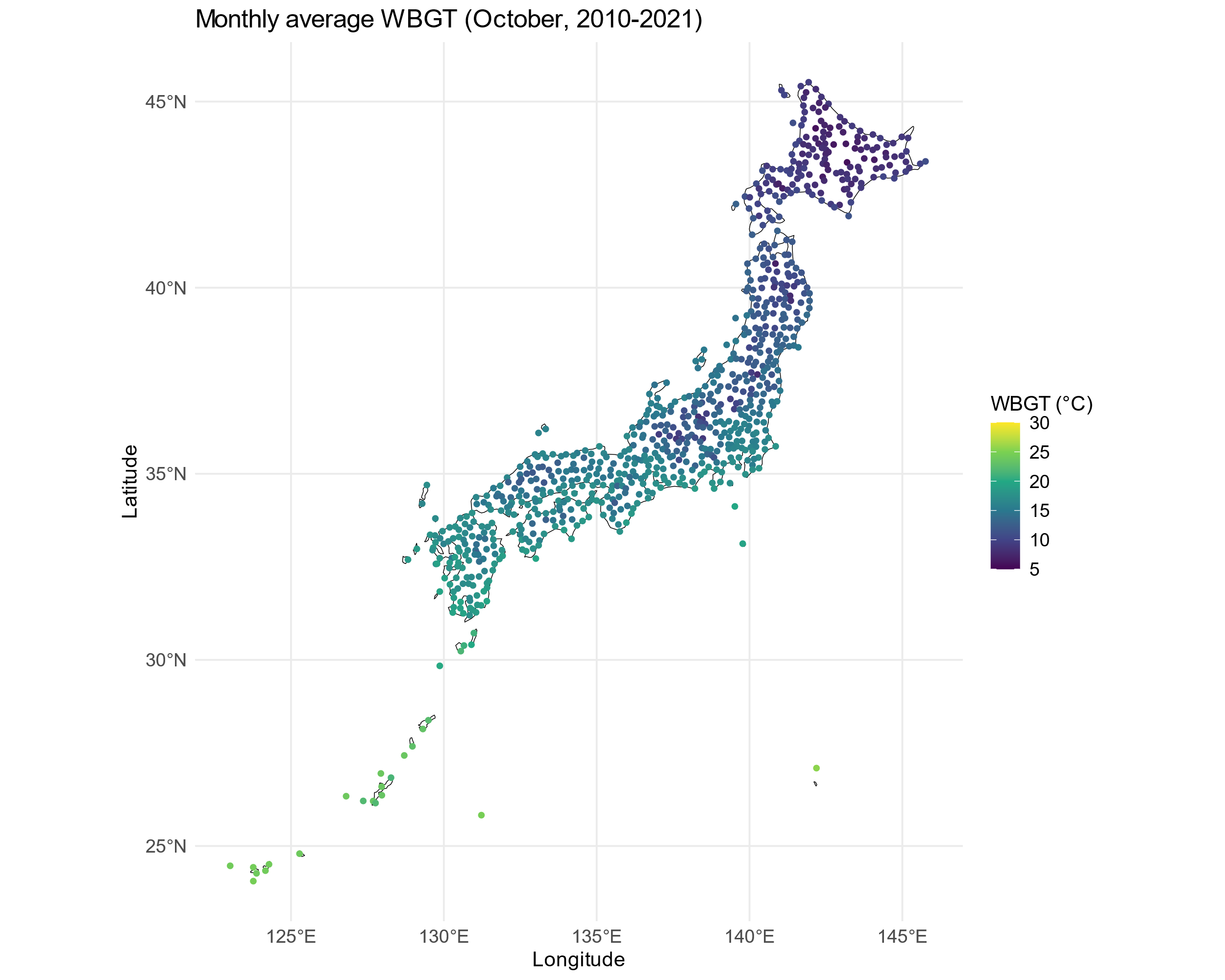 | 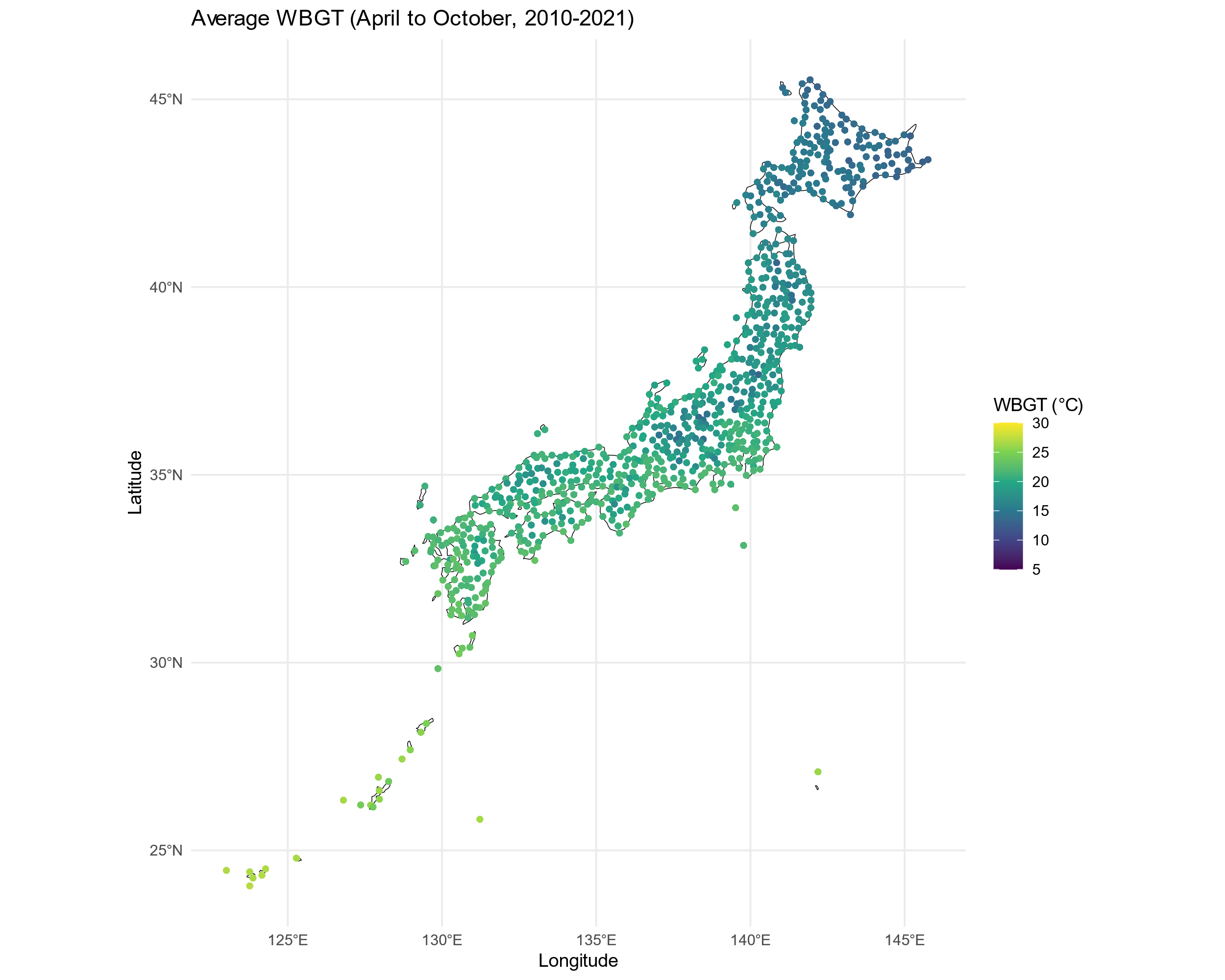 | Legend  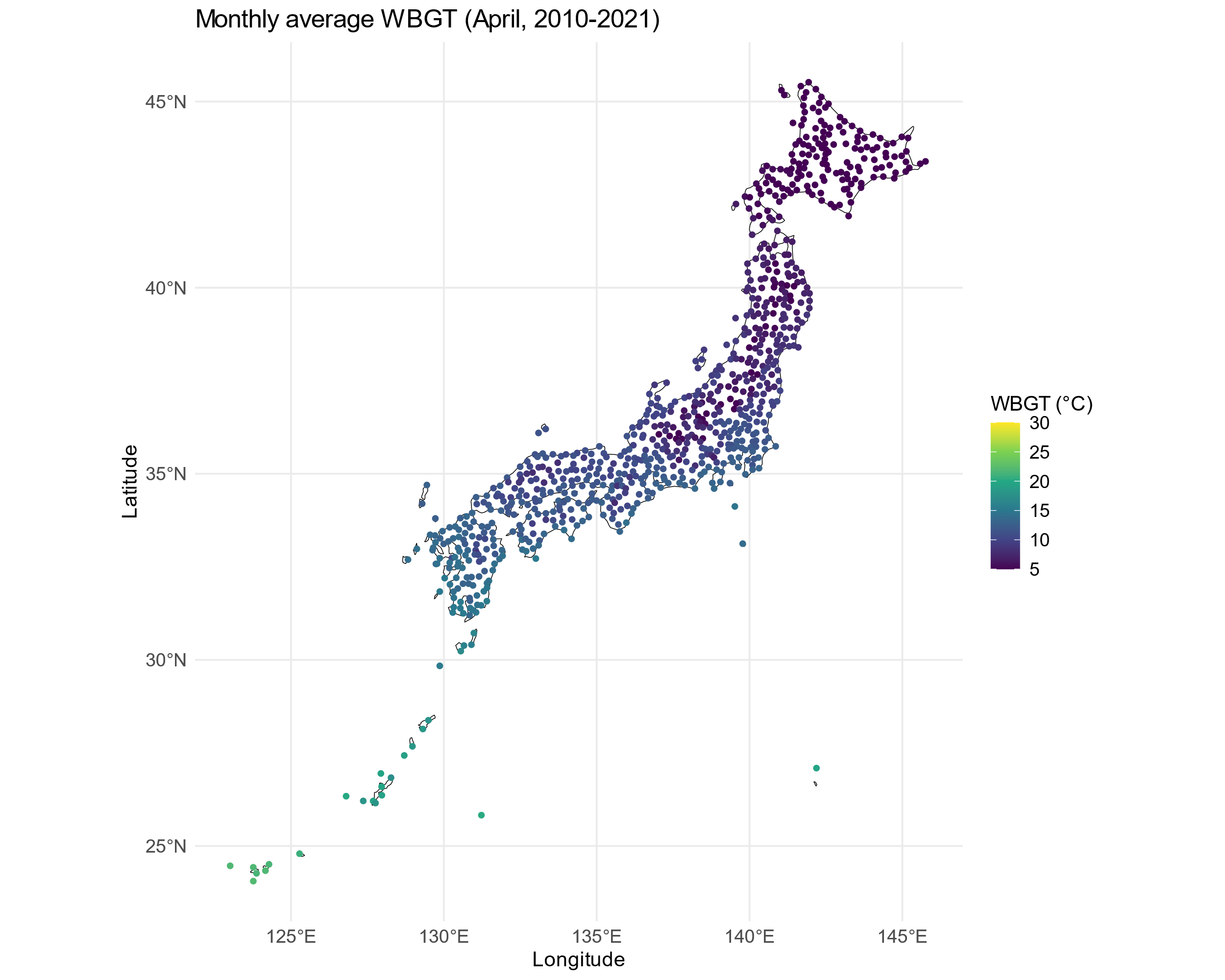 |
| 1. October | 1. May to October   (WBGT-Summer) |  |

Supplementary Fig. 1 Monthly average wet-bulb globe temperature (WBGT) value from April to October 2010~2021 and the average WBGT from May to October 2010~2021 (WBGT-Summer) of each site in Japan where WBGT is estimated by Ministry of the Environment, Japan

**
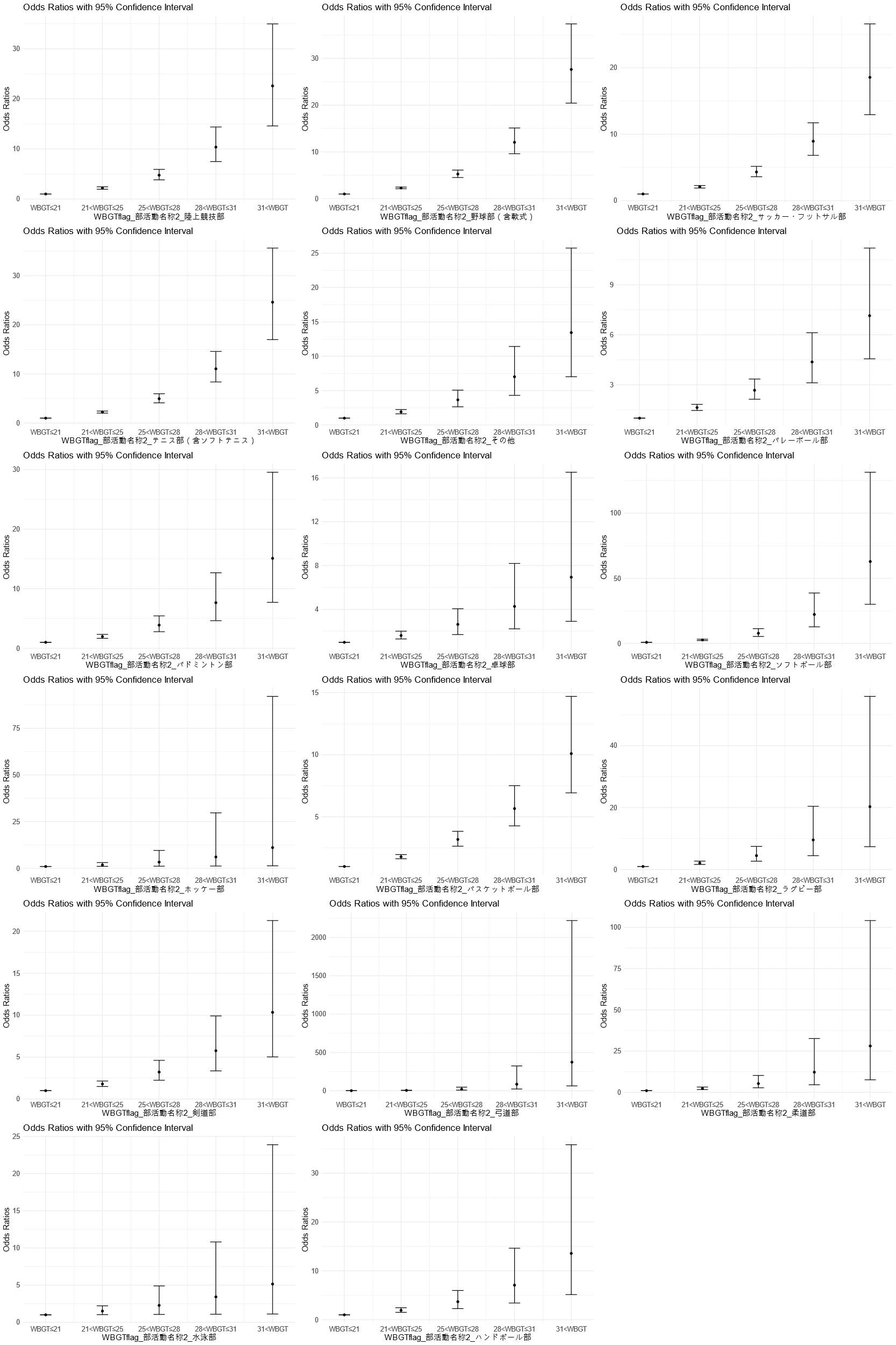
**

Supplementary Fig. 2 (1/2) Odds ratios and 95% confidence intervals for heat illness incidents in five wet-bulb globe temperature (WBGT) categories (odds ratio of 1 for WBGT≦21°C, stratified by club):

**- Top row: Track and field (left), Baseball (center), Football/Futsal (right)**

**- Middle row: Tennis (left), Other (center), Volleyball (right)**

**- Bottom row: Badminton (left), Table tennis (center), Softball (right)**

**
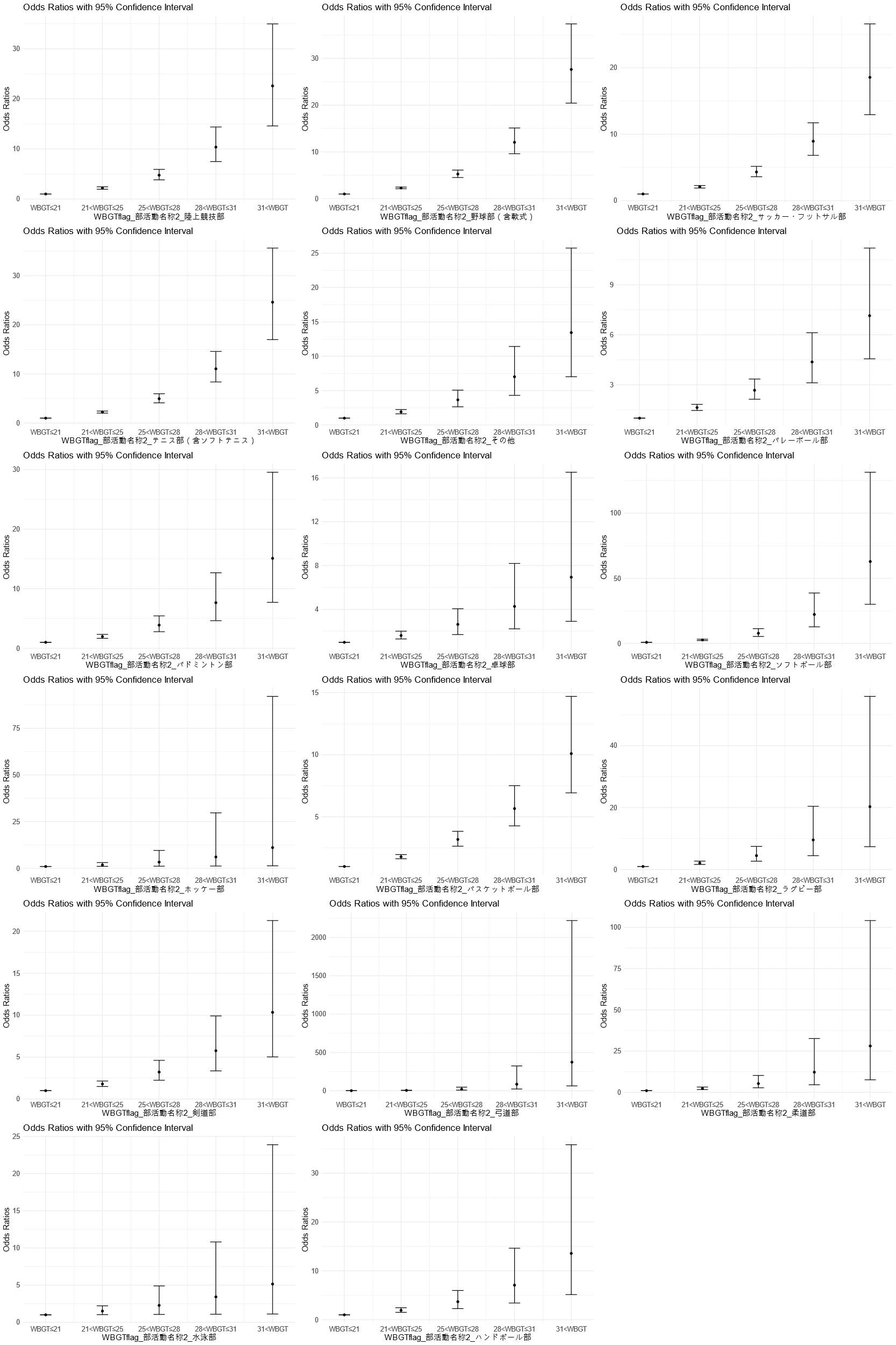
**

**
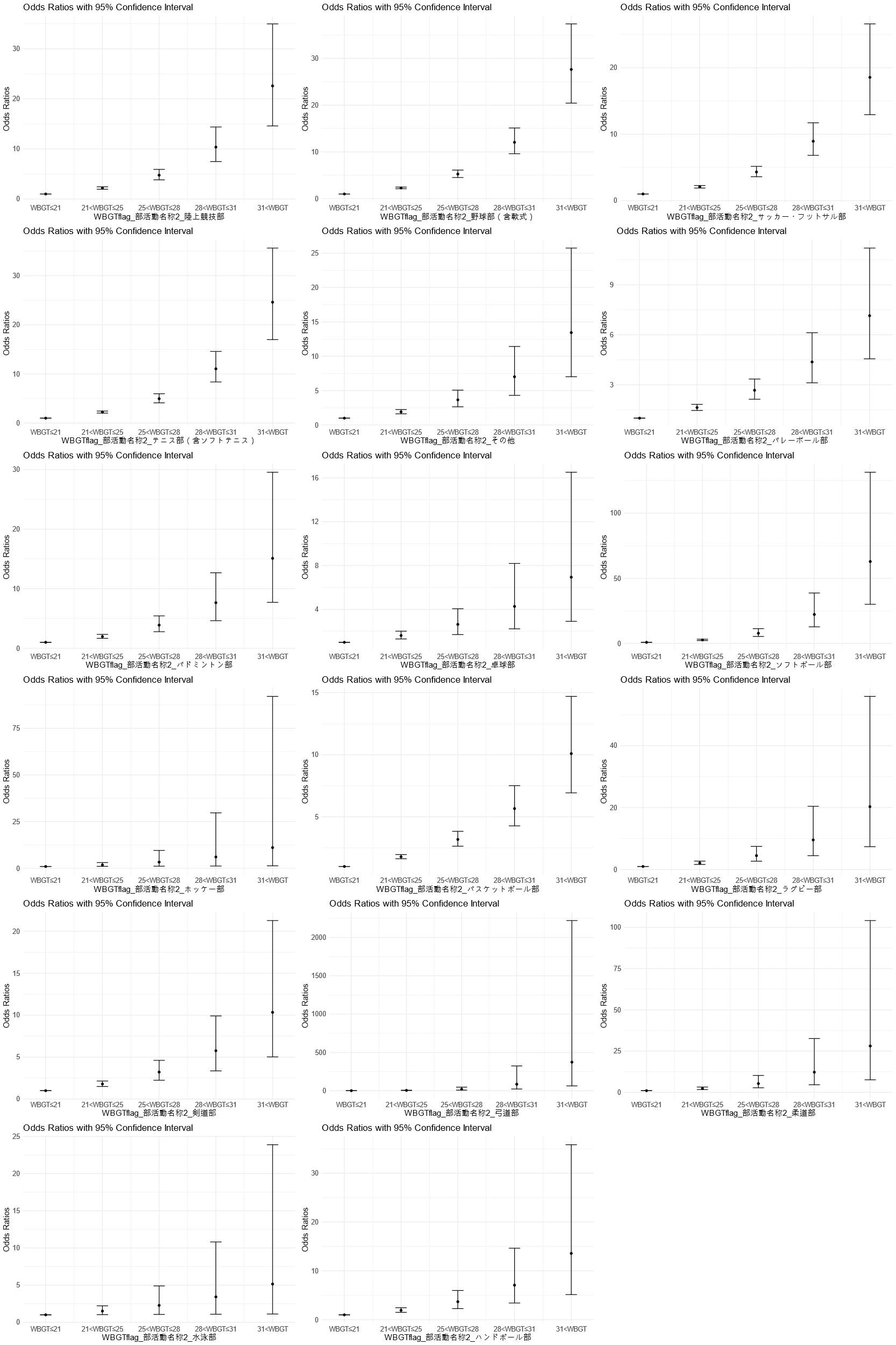
**

Supplementary Fig. 1 (2/2) Odds ratios and 95% confidence intervals for heat illness incidents in five wet-bulb globe temperature (WBGT) categories (odds ratio of 1 for WBGT≦21°C, stratified by club):

**- Top row: Field hockey (left), Basketball (center), Rugby (right)**

**- Middle row: Kendo (left), Kyudo (center), Judo (right)**

**- Bottom row: Swimming (left), Handball (center)**


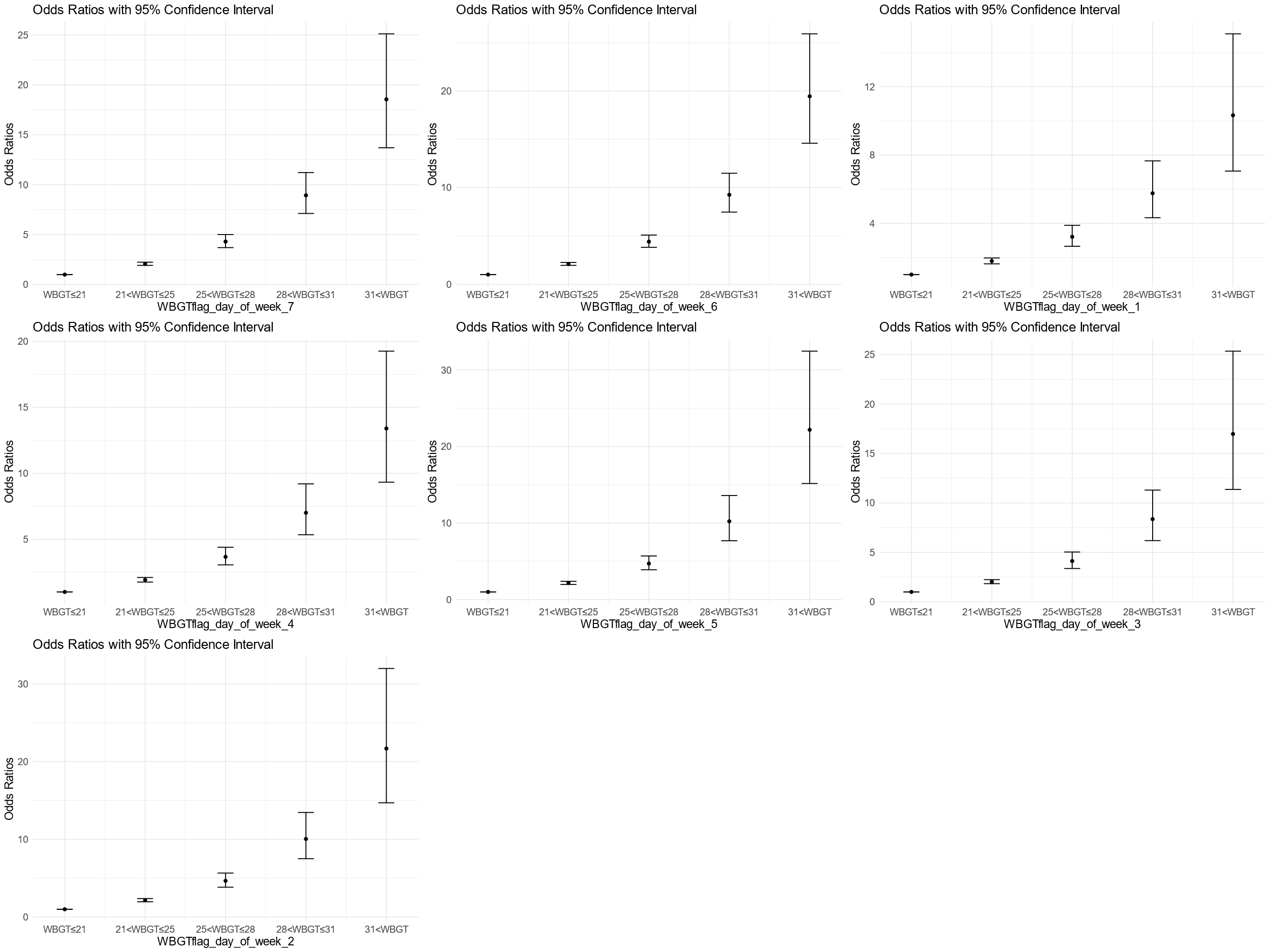
 Supplementary Fig. 3 Odds ratios and 95% confidence intervals for heat illness incidents in five wet-bulb globe temperature (WBGT) categories (odds ratio of 1 for WBGT≦21°C, stratified by day of week):

**- Top row: Sunday (left), Saturday (center), Monday (right)**

**- Middle row: Thursday (left), Friday (center), Wednesday (right)**

**- Bottom row: Tuesday (left)**


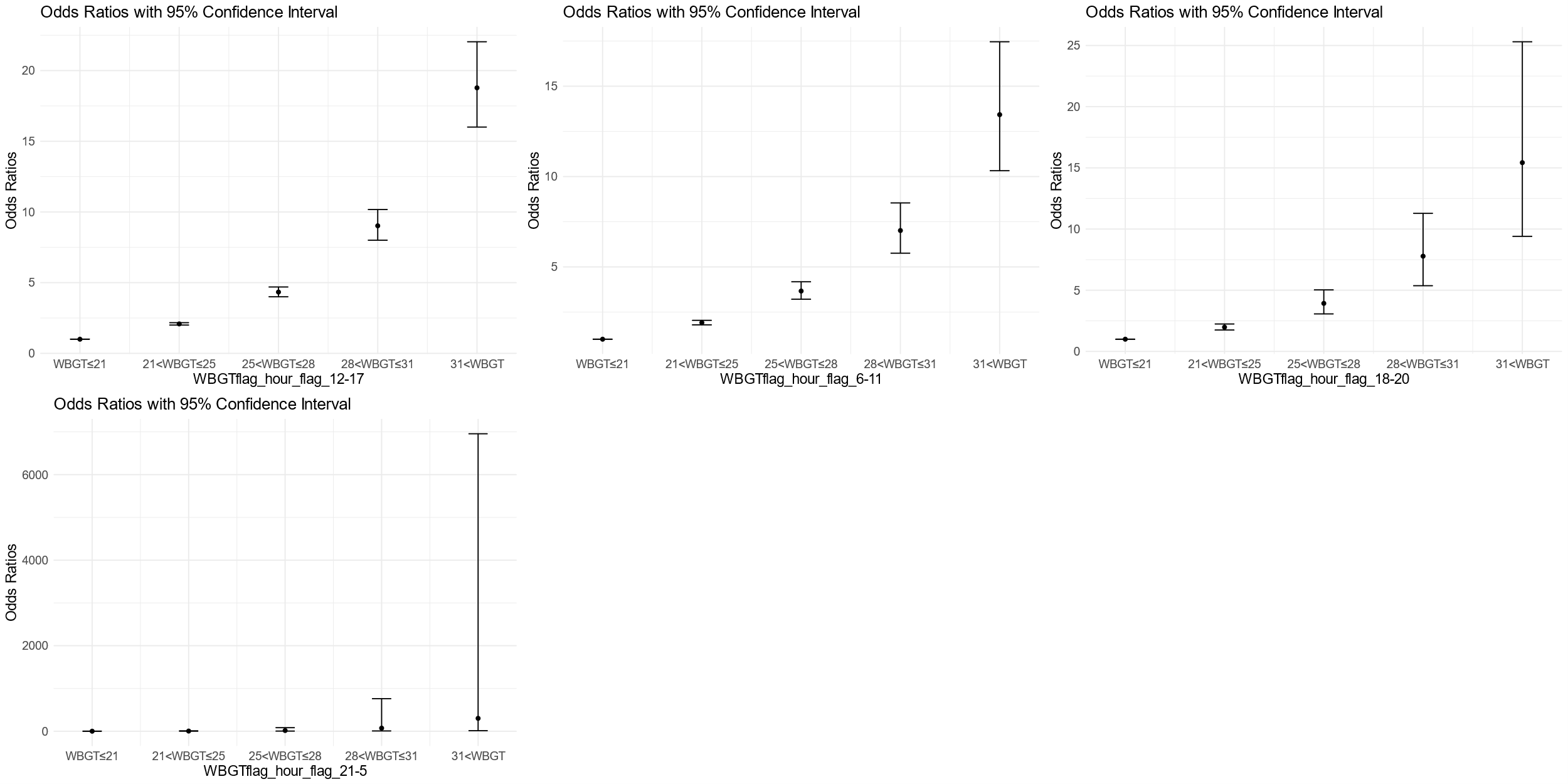
 Supplementary Fig. 4 Odds ratios and 95% confidence intervals for heat illness incidents in five wet-bulb globe temperature (WBGT) categories (odds ratio of 1 for WBGT≦21°C, stratified by hour)


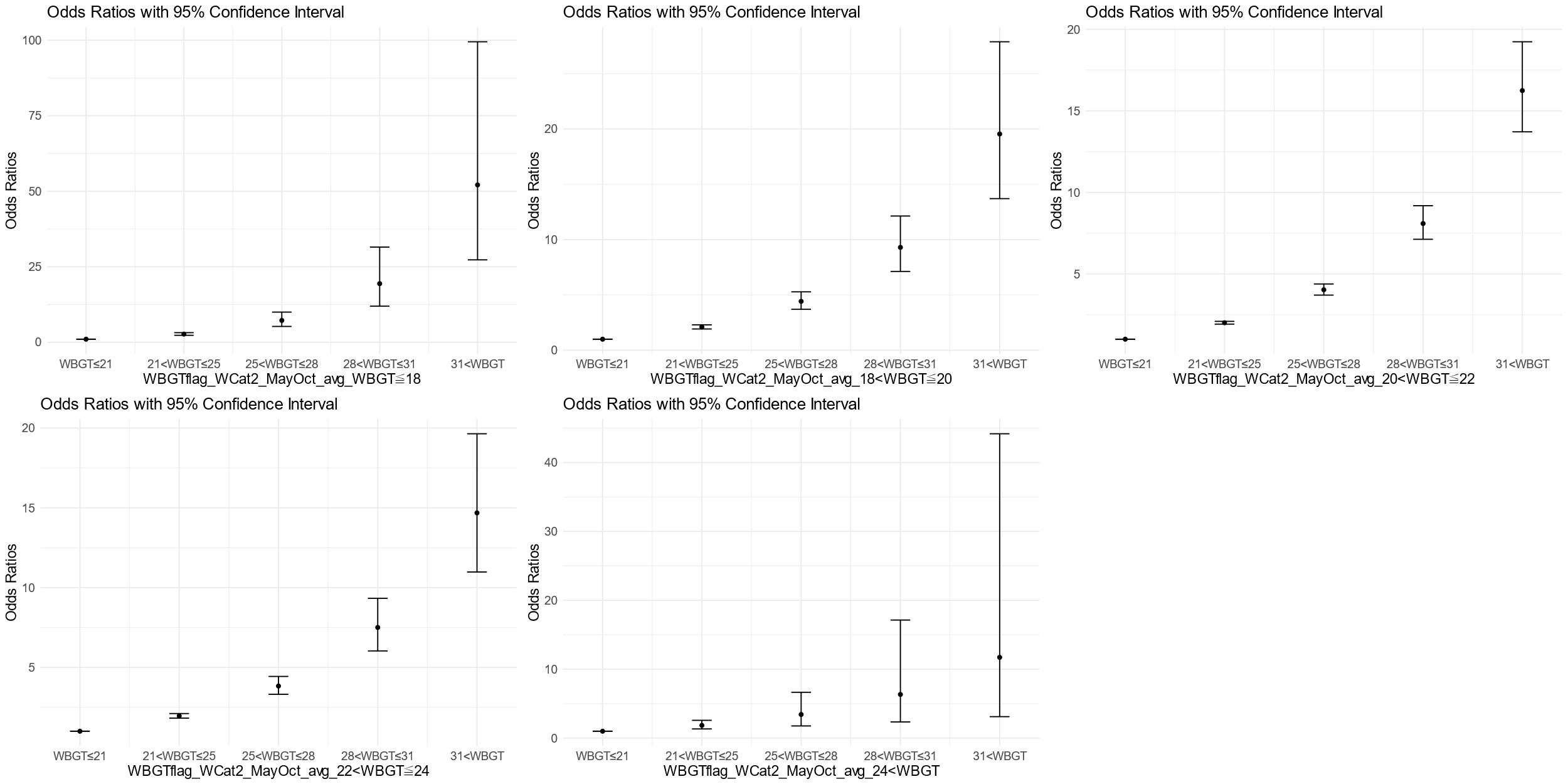
 Supplementary Fig. 5 Odds ratios and 95% confidence intervals for heat illness incidents in five wet-bulb globe temperature (WBGT) categories (odds ratio of 1 for WBGT≦21°C, stratified by average WBGT from May to October 2010~2021 (WBGT-Summer))


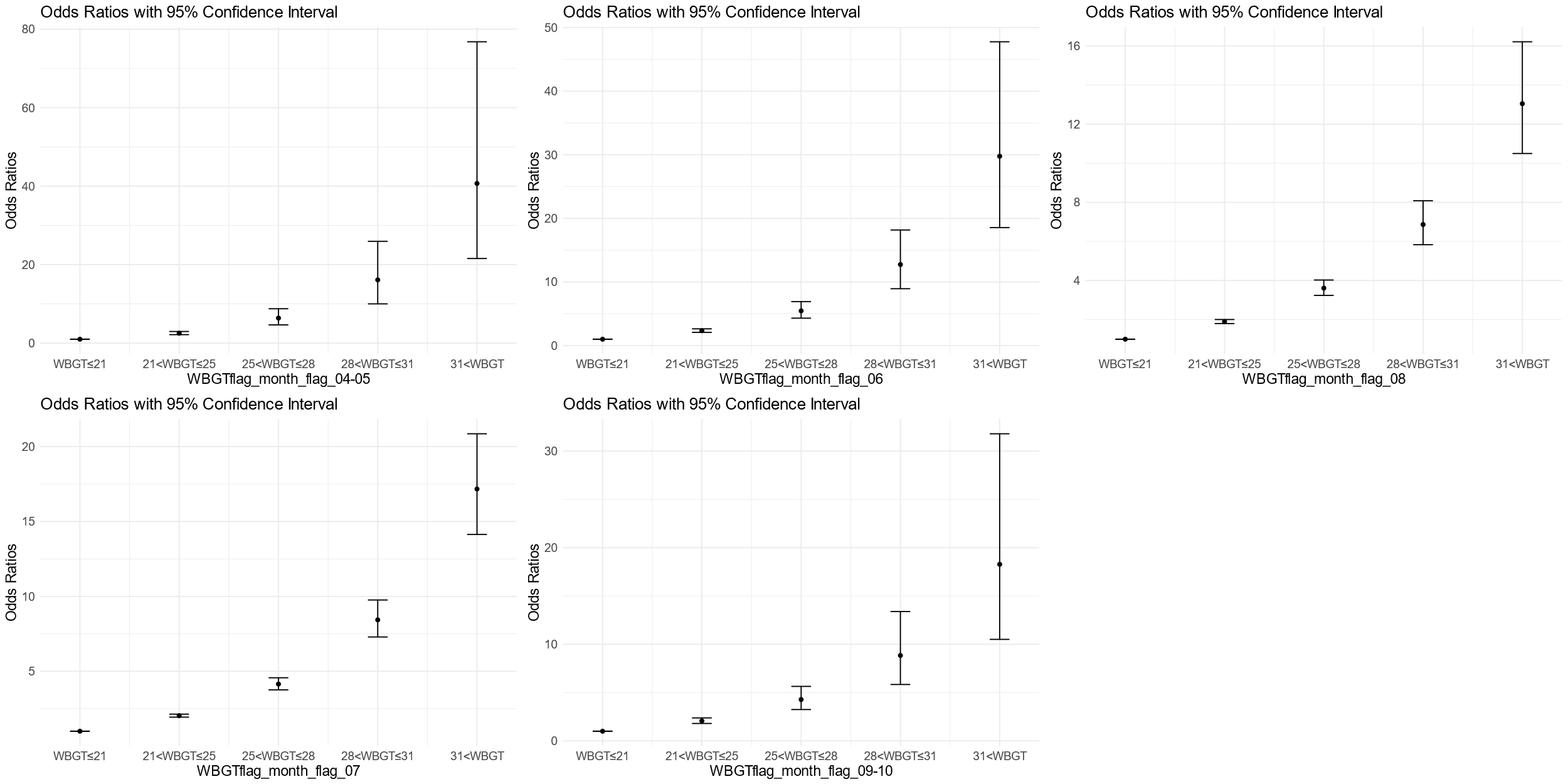
 Supplementary Fig. 6 Odds ratios and 95% confidence intervals for heat illness incidents in five wet-bulb globe temperature (WBGT) categories (odds ratio of 1 for WBGT≦21°C, stratified by month):

**- Top row: April to May (left), June (center), August (right)**

**- Bottom row: July (left), September to October (center)**

**
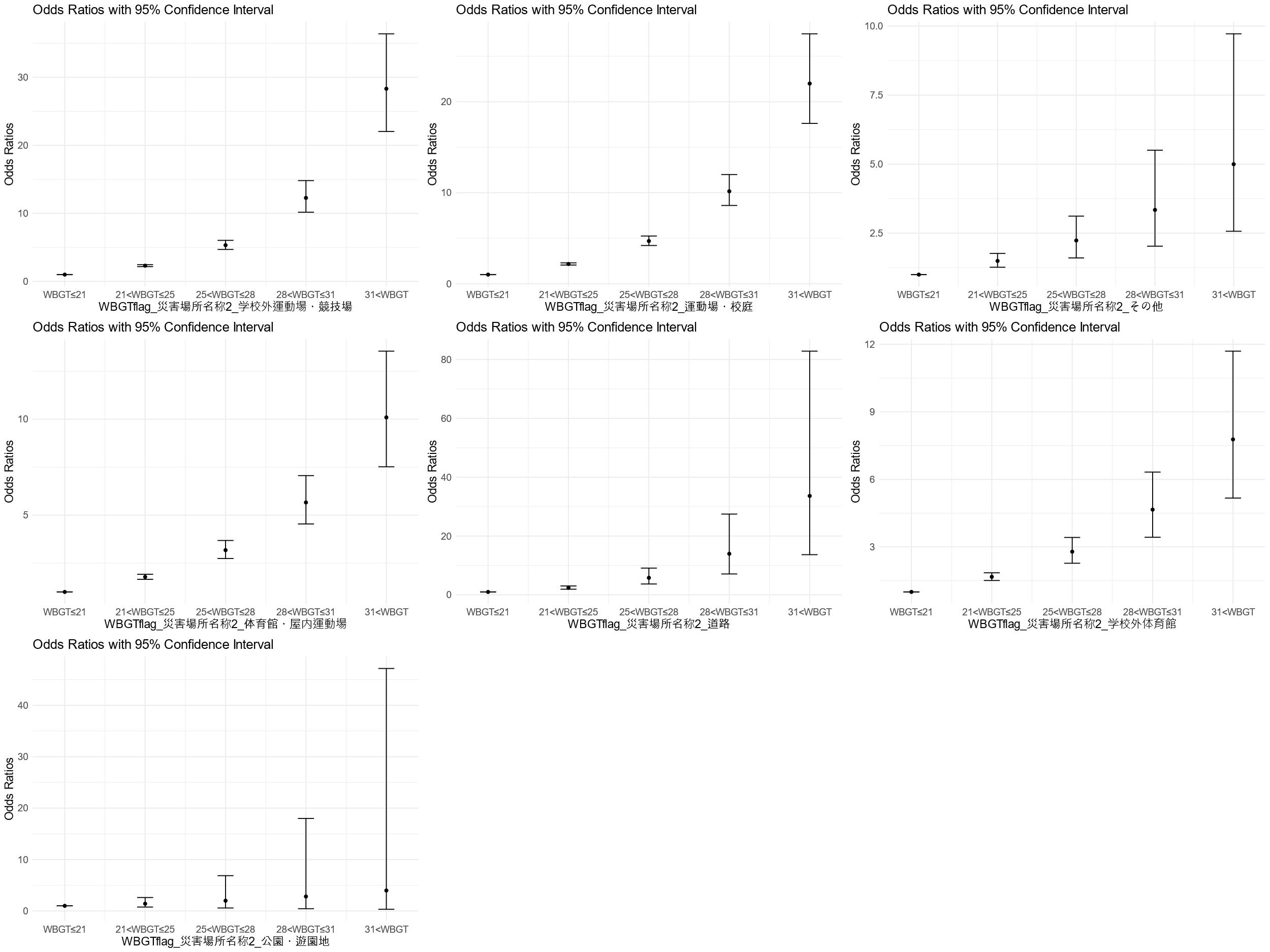
**

Supplementary Fig. 7 Odds ratios and 95% confidence intervals for heat illness incidents in five wet-bulb globe temperature (WBGT) categories (odds ratio of 1 for WBGT≦21°C, stratified by location):

**- Top row: Out-of-school playgrounds and stadiums (left), Playgrounds and schoolyards (center), Other (right)**

**- Middle row: Gymnasium/Indoor sports ground (left), Roads (center), Out-of-school sports halls (right)**

**- Bottom row: Parks and amusement parks (left)**

**
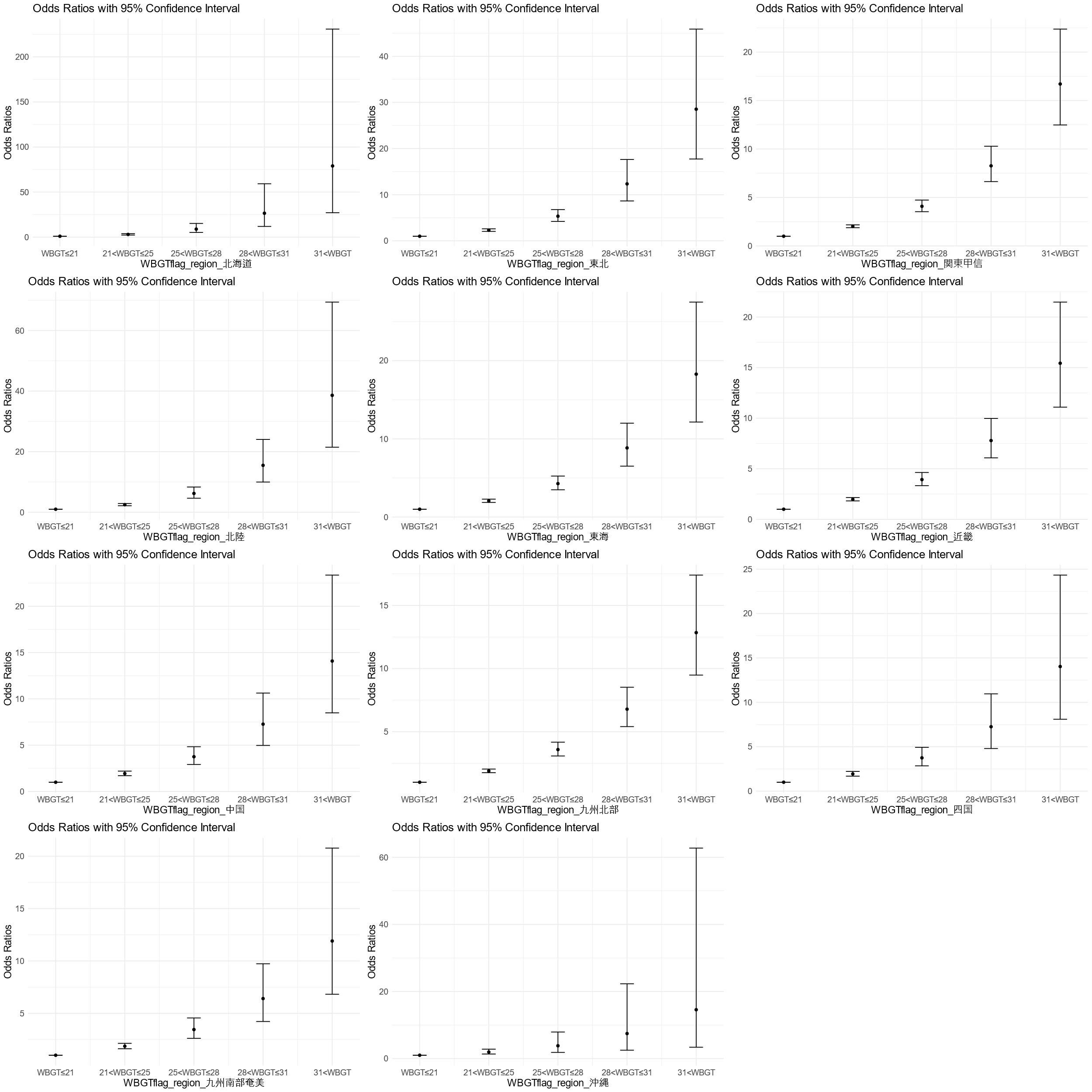
 Supplementary Fig. 8 Odds ratios and 95% confidence intervals for heat illness incidents in five wet-bulb globe temperature (WBGT) categories (odds ratio of 1 for WBGT≦21°C, stratified by region):**

**- Top row: Hokkaido (left), Tohoku (center), Kanto-Koshin (right)**

**- Upper-middle row: Hokuriku (left), Tokai (center), Kinki (right)**

**- Lower-middle row: Chugoku (left), Northern Kyushu (center), Shikoku (right)**

**- Bottom row: Southern Kyushu and Amami (left), Okinawa (center)**

**
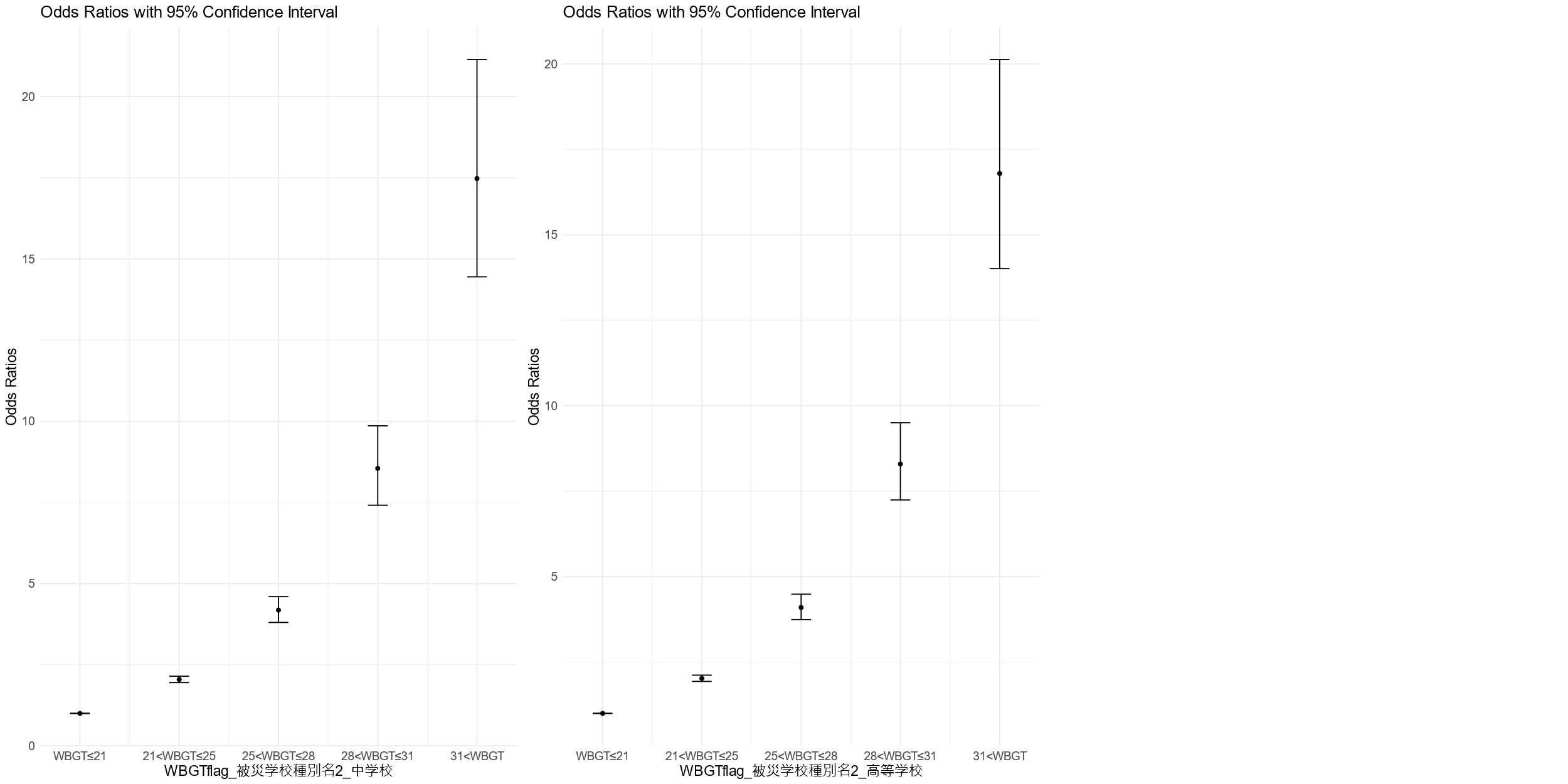
**

Supplementary Fig. 9 Odds ratios and 95% confidence intervals for heat illness incidents in five wet-bulb globe temperature (WBGT) categories (odds ratio of 1 for WBGT≦21°C, stratified by school):

**- Junior high school (left), High school (right)**

**
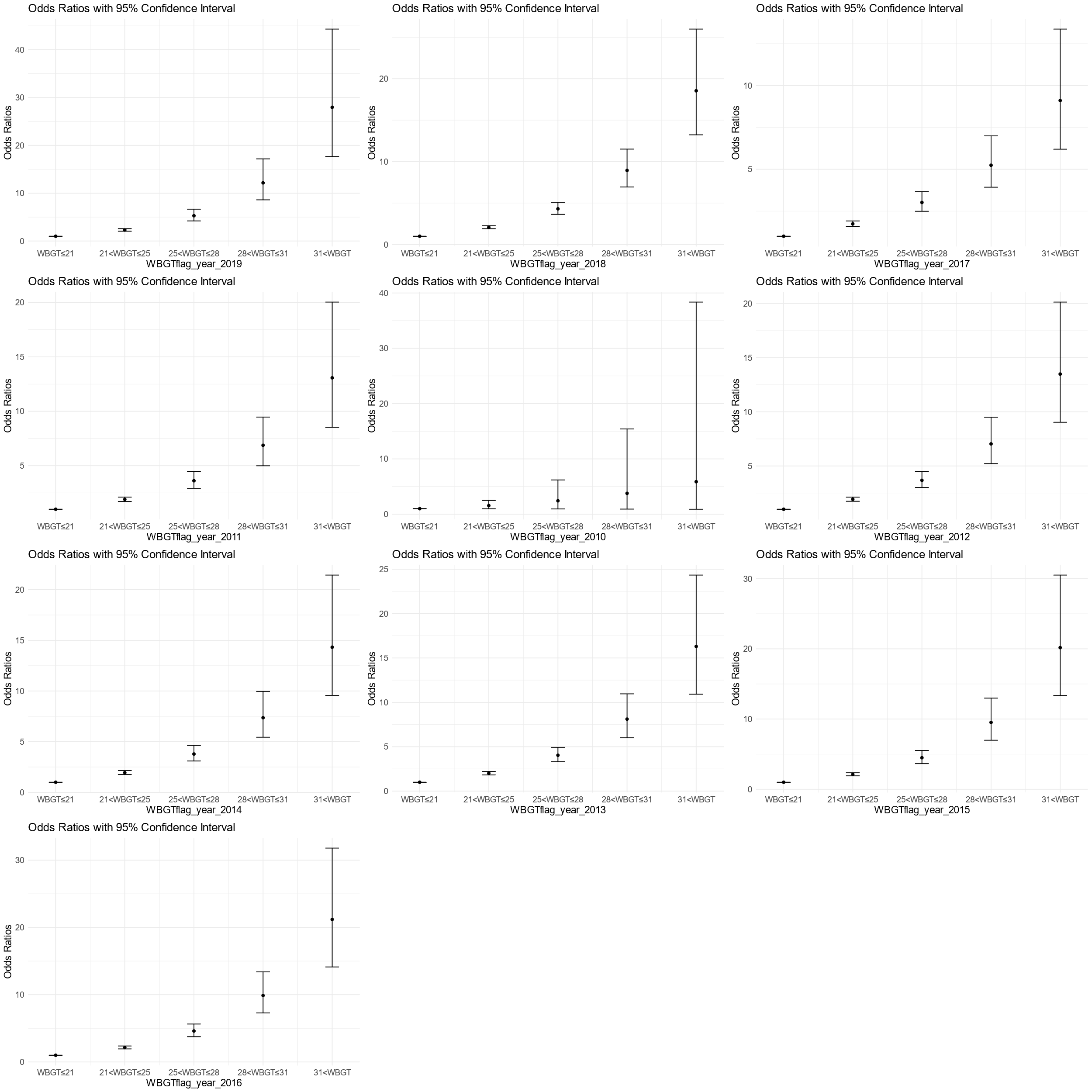
 Supplementary Fig. 10 Odds ratios and 95% confidence intervals for heat illness incidents in five wet-bulb globe temperature (WBGT) categories (odds ratio of 1 for WBGT≦21°C, stratified by year)**


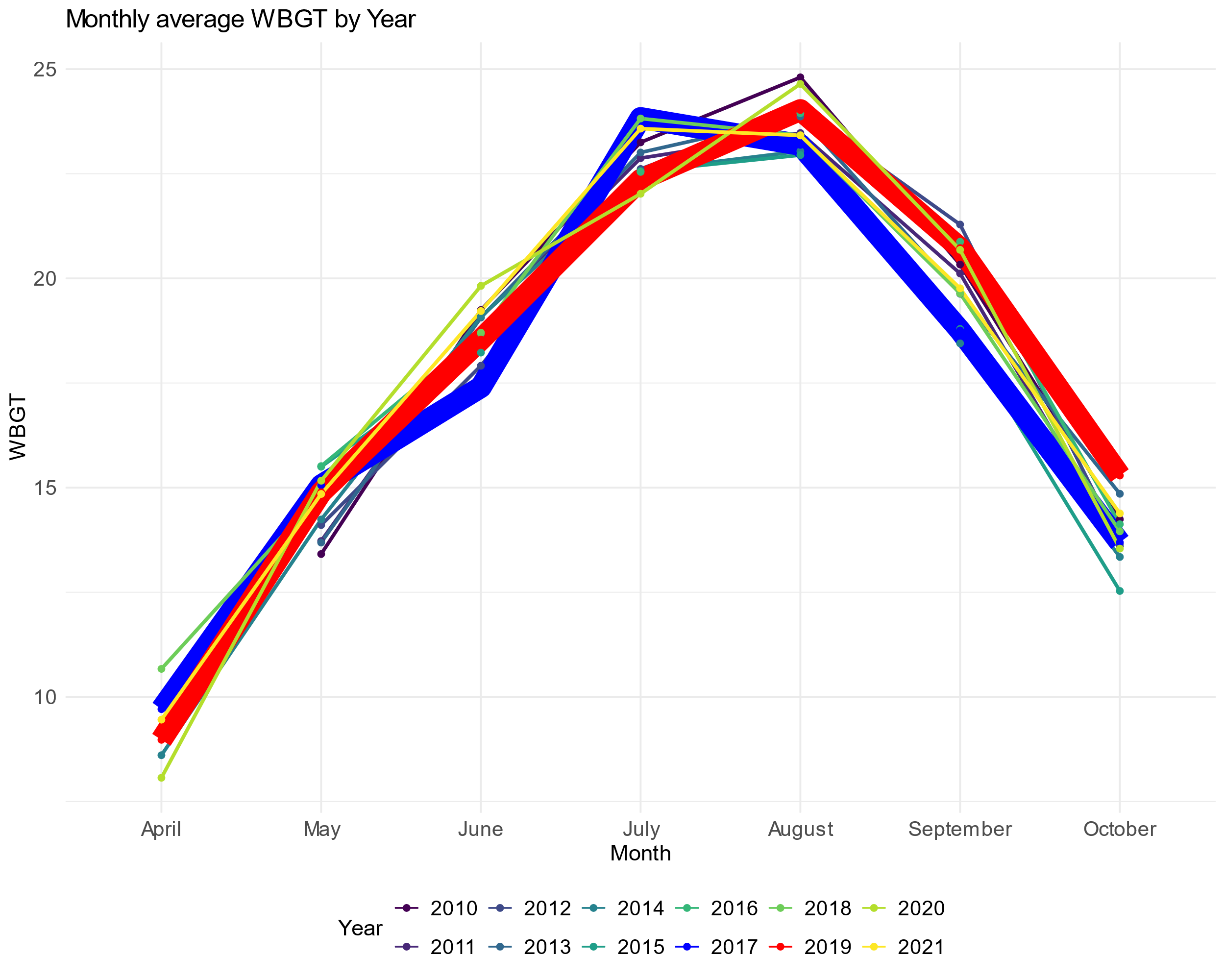
 Supplementary Fig. 11 Monthly mean wet-bulb globe temperature (WBGT) estimated by the Ministry of Environment from 2010 to 2021. Of the total 842 sites, values were averaged from 833 sites with data for all years and months. Lines for 2017 and 2019, where significant differences in odds ratios were found, are thicker.

Supplementary Table 1 Odds ratios for heat illness incidents by stratum (95% confidence intervals) and revised wet-bulb globe temperature thresholds (for the current category of 28<WBGT≦31℃ for ceasing strenuous exercise, with the odds ratio for WBGT≦21°C set as 1). The gray sections indicate nonsignificant strata.

| EV1 (group) | EV2 (stratification) | OR (95%CI) | Significance of models: Bonf. / B&H (p-values) ^a^ | Variables significantly different (p-value for Bonf.) ^b^ | Variables significantly different (p-value for B&H) ^c^ | Revised WBGT threshold ^d^ | Revised  p-value for Bonf. | n |
| --- | --- | --- | --- | --- | --- | --- | --- | --- |
| Club | Badminton | 7.66 (4.63-12.68) | T (<2.20e-16) / T (2.62e-16) | N/A | N/A | N/A | N/A | 2,807 |
|  | Baseball (incl. rubber-ball baseball) | 12.05 (9.61-15.11) | T (<2.20e-16) / T (2.62e-16) | Basketball (4.22e-5), Volleyball (9.80e-7) | Basketball (9.57e-4),  Table tennis (1.96e-2),  Volleyball (6.67e-5) | 25<WBGT≦28 | Basketball (0.649), Volleyball (0.327) | 14,110 |
|  | Basketball | 5.66 (4.27-7.51) | T (<2.20e-16) / T (2.62e-16) | N/A | N/A | N/A | N/A | 7,611 |
|  | Field hockey | 6.09 (1.25-29.72) | T (4.63e-5)/  T (4.92e-5) | N/A | N/A | N/A | N/A | 328 |
|  | Football/Futsal | 8.94 (6.82-11.71) | T (<2.20e-16) / T (2.62e-16) | N/A | Volleyball (9.25e-3) | 25<WBGT≦28 | N/A | 9,203 |
|  | Handball | 7.07 (3.42-14.64) | T (<2.20e-16) / T (2.62e-16) | N/A | N/A | N/A | N/A | 1,406 |
|  | Judo | 12.20 (4.57-32.60) | T (4.06e-8)  / T (4.52e-8) | N/A | N/A | N/A | N/A | 812 |
|  | Kendo | 5.77 (3.36-9.91) | T (<2.20e-16) / T (2.62e-16) | N/A | N/A | N/A | N/A | 2,365 |
|  | Kyudo (Japanese archery) | 84.90 (22.28-323.50) | T (4.16e-4)  / T (4.27e-4) | Basketball (1.04e-4),  Table tennis (8.21e-5),  Volleyball (2.50e-5) | Badminton (8.27e-3),  Baseball (2.51e-2),  Basketball (1.76e-3),  Football (9.25e-3),  Handball (9.88e-3),  Kendo (3.53e-3),  Rugby (2.62e-2),  Swimming (4.08e-3),  Table tennis (1.59e-3),  Tennis (1.97e-2),  Track and field (1.79e-2),  Volleyball (8.50e-4),  Other (5.83e-3) | 25<WBGT≦28 | Basketball (1.01e-2),  Table tennis (7.40e-3),  Volleyball (2.25e-3) | 453 |
|  | Rugby | 9.57 (4.48-20.44) | T (4.78e-15)  / T (5.60e-15) | N/A | N/A | N/A | N/A | 1,316 |
|  | Softball | 22.34 (12.86-38.80) | T (<2.20e-16) / T (2.62e-16) | Basketball (1.42e-5),  Table tennis (1.45e-4),  Volleyball (7.66e-7) | Badminton (2.52e-2),  Basketball (6.45e-4),  Football (1.97e-2),  Kendo (5.83e-3),  Table tennis (2.19e-3),  Volleyball (6.67e-5),  Other (1.40e-2) | 25<WBGT≦28 | Basketball (0.154),  Table tennis (0.105),  Volleyball (1.93e-2) | 2,775 |
|  | Swimming^e^ | 3.41 (1.08-10.80) | T (2.90e-13)  / T (3.34e-13) | N/A | N/A | N/A | N/A | 648 |
|  | Table tennis | 4.28 (2.23-8.19) | T (6.21e-10)  / T (7.04e-10) | N/A | N/A | N/A | N/A | 1,469 |
|  | Tennis (incl. soft tennis) | 11.05 (8.37-14.58) | T (<2.20e-16) / T (2.62e-16) | Volleyball (3.18e-5) | Basketball (8.27e-3),  Table tennis (3.86e-2),  Volleyball (8.66e-4) | 25<WBGT≦28 | Volleyball (0.520) | 9,484 |
|  | Track and field | 10.36 (7.46-14.38) | T (<2.20e-16) / T (2.62e-16) | N/A | Basketball (2.89e-2),  Volleyball (3.98e-3) | 25<WBGT≦28 | N/A | 6,627 |
|  | Volleyball | 4.37 (3.12-6.12) | T (<2.20e-16) / T (2.62e-16) | N/A | N/A | N/A | N/A | 5,204 |
|  | Other | 7.02 (4.32-11.48) | T (<2.20e-16) / T (2.62e-16) | N/A | N/A | N/A | N/A | 2,909 |
| Day of Week | Monday^e^ | 5.76 (4.33-7.66) | T (<2.20e-16) / T (2.62e-16) | N/A | N/A | N/A | N/A | 8,658 |
|  | Tuesday | 10.04 (7.50-13.44) | T (<2.20e-16) / T (2.62e-16) | N/A | N/A | N/A | N/A | 8,337 |
|  | Wednesday | 8.37 (6.19-11.30) | T (<2.20e-16) / T (2.62e-16) | N/A | N/A | N/A | N/A | 8,641 |
|  | Thursday | 7.00 (5.33-9.19) | T (<2.20e-16) / T (2.62e-16) | N/A | N/A | N/A | N/A | 8,480 |
|  | Friday | 10.22 (7.69-13.60) | T (<2.20e-16) / T (2.62e-16) | N/A | N/A | N/A | N/A | 8,202 |
|  | Saturday | 9.26 (7.47-11.49) | T (<2.20e-16) / T (2.62e-16) | N/A | N/A | N/A | N/A | 14,198 |
|  | Sunday | 8.94 (7.12-11.22) | T (<2.20e-16) / T (2.62e-16) | N/A | N/A | N/A | N/A | 13,011 |
| Hour | 06-11^e^ | 7.01 (5.76-8.54) | T (<2.20e-16) / T (2.62e-16) | N/A | N/A | N/A | N/A | 18,714 |
|  | 12-17 | 9.02 (8.00-10.17) | T (<2.20e-16) / T (2.62e-16) | N/A | N/A | N/A | N/A | 43,807 |
|  | 18-20 | 7.78 (5.37-11.28) | T (<2.20e-16) / T (2.62e-16) | N/A | N/A | N/A | N/A | 6,609 |
|  | 21-5 | 72.34 (6.87-761.58) | T (2.21e-5)  / T (2.38e-5) | N/A | N/A | N/A | N/A | 397 |
| WBGT-Summer | WBGT≦18 | 19.39 (11.93-31.50) | T (<2.20e-16) / T (2.62e-16) | 20<WBGT≦22 (6.38e-4),  22<WBGT≦24 (4.68e-4) | 18<WBGT≦20 (3.08e-2),  20<WBGT≦22 (3.19e-3),  22<WBGT≦24 (3.19e-3) | 25<WBGT≦28 | 20<WBGT≦22 (0.520),  22<WBGT≦24 (0.846) | 3,241 |
|  | 18<WBGT≦20 | 9.30 (7.12-12.13) | T (<2.20e-16) / T (2.62e-16) | N/A | N/A | N/A | N/A | 8,603 |
|  | 20<WBGT≦22 | 8.09 (7.13-9.19) | T (<2.20e-16) / T (2.62e-16) | N/A | N/A | N/A | N/A | 41,734 |
|  | 22<WBGT≦24 | 7.50 (6.03-9.33) | T (<2.20e-16) / T (2.62e-16) | N/A | N/A | N/A | N/A | 14,894 |
|  | 24<WBGT^e^ | 6.33 (2.34-17.13) | T (2.18e-4)  / T (2.28e-4) | N/A | N/A | N/A | N/A | 1,055 |
| Month | April-May | 16.11 (10.01-25.94) | T (<2.20e-16)  / T (2.62e-16) | August (8.96e-4) | July  (3.63e-2),  August (8.96e-3) | 25<WBGT≦28 | August (0.687) | 3,860 |
|  | June | 12.74 (8.94-18.17) | T (<2.20e-16) / T (2.62e-16) | August (1.89e-3) | August (9.46e-3) | 25<WBGT≦28 | August (0.117) | 5,616 |
|  | July | 8.43 (7.29-9.76) | T (<2.20e-16) / T (2.62e-16) | N/A | N/A | N/A | N/A | 31,614 |
|  | August  ^e^ | 6.86 (5.83-8.08) | T (<2.20e-16) / T (2.62e-16) | N/A | N/A | N/A | N/A | 24,509 |
|  | September-October | 8.84 (5.84-13.39) | T (<2.20e-16) / T (2.62e-16) | N/A | N/A | N/A | N/A | 3,928 |
| Region | Hokkaido | 26.50 (11.86-59.22) | T (<2.20e-16) / T (2.62e-16) | N/A | Kanto-Koshin (6.14e-3),  Kinki (4.33e-3),  Chugoku (4.36e-3),  Shikoku (4.95e-3),  Northern Kyushu (1.40e-3),  Southern Kyushu and Amami (2.14e-3) | 25<WBGT≦28 | N/A | 1,283 |
|  | Tohoku | 12.34 (8.64-17.63) | T (<2.20e-16) / T (2.62e-16) | N/A | Northern Kyushu (3.75e-2) | 25<WBGT≦28 | N/A | 5,221 |
|  | Kanto-Koshin | 8.26 (6.64-10.29) | T (<2.20e-16) / T (2.62e-16) | N/A | N/A | N/A | N/A | 12,912 |
|  | Hokuriku | 15.89 (10.19-24.76) | T (<2.20e-16) / T (2.62e-16) | N/A | Kinki (4.16e-2),  Northern Kyushu (3.75e-2),  Southern Kyushu and Amami (3.75e-2) | 25<WBGT≦28 | N/A | 3,719 |
|  | Tokai | 8.84 (6.51-12.00) | T (<2.20e-16) / T (2.62e-16) | N/A | N/A | N/A | N/A | 7,746 |
|  | Kinki | 7.79 (6.08-9.98) | T (<2.20e-16) / T (2.62e-16) | N/A | N/A | N/A | N/A | 11,624 |
|  | Chugoku | 7.27 (4.97-10.63) | T (<2.20e-16) / T (2.62e-16) | N/A | N/A | N/A | N/A | 5,156 |
|  | Shikoku | 7.25 (4.80-10.96) | T (<2.20e-16) / T (2.62e-16) | N/A | N/A | N/A | N/A | 4,214 |
|  | Northern Kyushu | 6.79 (5.40-8.52) | T (<2.20e-16) / T (2.62e-16) | N/A | N/A | N/A | N/A | 12,818 |
|  | Southern Kyushu and Amami^e^ | 6.41 (4.22-9.73) | T (<2.20e-16) / T (2.62e-16) | N/A | N/A | N/A | N/A | 3,877 |
|  | Okinawa | 7.46 (2.49-22.30) | T (1.07e-5)  / T (1.17e-5) | N/A | N/A | N/A | N/A | 957 |
| Location | Gymnasium/Indoor sports ground | 5.66 (4.54-7.06) | T (<2.20e-16) / T (2.62e-16) | N/A | N/A | N/A | N/A | 13,122 |
|  | Out-of-school playgrounds and stadiums | 12.28 (10.17-14.82) | T (<2.20e-16) / T (2.62e-16) | Gymnasium/Indoor sports ground (1.69e-7),  Out-of-school sports halls  (1.24e-7),  Other  (1.73e-6) | Gymnasium/Indoor sports ground (1.77e-6),  Out-of-school sports halls  (1.77e-6),  Other (1.21e-5) | 25<WBGT≦28 | Gymnasium/Indoor sports ground (0.631),  Out-of-school sports halls (0.429),  Other (7.63e-2) | 19,752 |
|  | Out-of-school sports halls | 4.66 (3.43-6.33) | T (<2.20e-16) / T (2.62e-16) | N/A | N/A | N/A | N/A | 6,207 |
|  | Parks and amusement parks  ^e^ | 2.82 (0.44-17.99) | T (4.20e-4)  / T (4.27e-4) | N/A | N/A | N/A | N/A | 337 |
|  | Playgrounds and schoolyards | 10.16 (8.60-12.00) | T (<2.20e-16) / T (2.62e-16) | Gymnasium/Indoor sports ground (3.42e-5),  Out-of-school sports halls  (1.15e-5),  Other  (3.43e-5) | Gymnasium/Indoor sports ground (1.20e-4),  Out-of-school sports halls  (6.05e-5),  Other (1.20e-4) | 25<WBGT≦28 | Gymnasium/Indoor sports ground (0.135),  Out-of-school sports halls (0.965),  Other (0.194) | 25,615 |
|  | Roads | 13.97 (7.11-27.46) | T (<2.20e-16) / T (2.62e-16) | Other  (8.48e-4) | Gymnasium/Indoor sports ground (2.99e-2),  Out-of-school sports halls  (9.75e-3),  Other (2.54e-3) | 25<WBGT≦28 | Other (0.108) | 1,752 |
|  | Other | 3.34 (2.03-5.50) | T (<2.20e-16) / T (2.62e-16) | N/A | N/A | N/A | N/A | 2,742 |
| School | Junior high school | 8.55 (7.41-9.86) | T (<2.20e-16) / T (2.62e-16) | N/A | N/A | N/A | N/A | 32,616 |
|  | High school  ^e^ | 8.30 (7.24-9.50) | T (<2.20e-16) / T (2.62e-16) | N/A | N/A | N/A | N/A | 36,911 |
| Year | 2010  ^e^ | 3.77 (0.92-15.41) | F (6.67e-3)  / T (6.67e-3) | N/A | N/A | N/A | N/A | 390 |
|  | 2011 | 6.88 (4.99-9.47) | T (<2.20e-16) / T (2.62e-16) | N/A | N/A | N/A | N/A | 7,002 |
|  | 2012 | 7.04 (5.21-9.51) | T (<2.20e-16) / T (2.62e-16) | N/A | N/A | N/A | N/A | 7,703 |
|  | 2013 | 8.11 (6.01-10.96) | T (<2.20e-16) / T (2.62e-16) | N/A | N/A | N/A | N/A | 7,913 |
|  | 2014 | 7.36 (5.44-9.96) | T (<2.20e-16) / T (2.62e-16) | N/A | N/A | N/A | N/A | 6,607 |
|  | 2015 | 9.52 (6.98-12.98) | T (<2.20e-16) / T (2.62e-16) | N/A | N/A | N/A | N/A | 7,260 |
|  | 2016 | 9.88 (7.29-13.39) | T (<2.20e-16) / T (2.62e-16) | N/A | N/A | N/A | N/A | 7,095 |
|  | 2017 | 5.24 (3.93-6.99) | T (<2.20e-16) / T (2.62e-16) | N/A | N/A | N/A | N/A | 7,665 |
|  | 2018 | 8.94 (6.94-11.51) | T (<2.20e-16) / T (2.62e-16) | N/A | N/A | N/A | N/A | 11,283 |
|  | 2019 | 12.16 (8.61-17.18) | T (<2.20e-16) / T (2.62e-16) | 2017  (2.45e-4) | 2017  (1.10e-2) | 25<WBGT≦28 | 2017 (0.962) | 6,609 |

Abbreviations: Bonf., Bonferroni’s method; B&H, Benjamini-Hochberg’s method; CI, confidence interval; EV, explanatory variable; OR, odds ratio; T, true; F, false; WBGT, wet-bulb globe temperature (℃), WBGT-Incident, the nearest hour WBGT at the time of heat illness incident, WBGT-Summer, average WBGT from May to October; α: significance levels.

^a^ α of models: 7.35e-4 (Bonf.), 0.05 (B&H). When calculating p-values for the B&H method, those with <2.20e-16 are considered as 2.20e-16 in the R program.

^b^ α of differences from other EV2s in the same EV1 (Bonf.): Club, 1.84e-4; Day of Week, 1.19e-3; Hour, 4.17e-3; Average WBGT from May to October, 2.50e-3; Month, 2.50e-3; Location, 1.19e-3; School, 2.50e-3; Year, 5.56e-4.

^c^ α of differences from other EV2s in same EV1 (B&H): 0.05.

^d^ Each Revised WBGT threshold was set so that if there was a significant difference in both Bonf. and B&H, the significant difference in Bonf. was eliminated, and if there was a significant difference only in the B&H, it was lowered by one category.

^e^ These EV2s were excluded from the comparison because their mean ORs were smaller than those of any of the EV2s for the same EV1.

Supplementary Table 2 Odds ratios for heat illness incidents by stratum (95% confidence intervals) and revised wet-bulb globe temperature thresholds (for the current category of 31℃ < WBGT for ceasing all exercise, with the odds ratio or WBGT < 21℃ set as 1) and revised p-value: Significance of models, variables significantly different, and sample size n are the same as in Supplementary Table 1.

| EV 1 (group) | EV 2 (stratification) | OR (95%CI) | Revised WBGT threshold | Revised p-value  for Bonf. |
| --- | --- | --- | --- | --- |
| Club | Badminton | 15.10  (7.72-29.56) | N/A | N/A |
|  | Baseball (incl. rubber-ball baseball) | 27.64  (20.44-37.37) | 28<WBGT≦31 | Basketball (0.428),  Volleyball (4.18e-2) |
|  | Basketball | 10.09  (6.93-14.70) | N/A | N/A |
|  | Field hockey | 11.11  (1.34-92.05) | N/A | N/A |
|  | Football/Futsal | 18.55  (12.94-26.59) | 28<WBGT≦31 | N/A |
|  | Handball | 13.57  (5.14-35.82) | N/A | N/A |
|  | Judo | 28.09  (7.58-104.15) | N/A | N/A |
|  | Kendo | 10.34  (5.02-21.28) | N/A | N/A |
|  | Kyudo (Japanese archery) | 373.16  (62.70-2220.78) | 28<WBGT≦31 | Basketball (2.66e-3),  Table tennis (2.08e-3),  Volleyball (5.88e-4) |
|  | Rugby | 20.32  (7.39-55.88) | N/A | N/A |
|  | Softball | 62.92  (30.14-131.34) | 28<WBGT≦31 | Basketball (1.97e-2),  Table tennis (2.58e-2),  Volleyball (1.70e-3) |
|  | Swimming^a^ | 5.14  (1.11-23.89) | N/A | N/A |
|  | Table tennis | 6.94  (2.92-16.52) | N/A | N/A |
|  | Tennis (incl. soft tennis) | 24.60  (16.99-35.63) | 28<WBGT≦31 | Volleyball (0.106) |
|  | Track and field | 22.58  (14.59-34.96) | 28<WBGT≦31 | N/A |
|  | Volleyball | 7.15  (4.56-11.20) | N/A | N/A |
|  | Other | 13.45  (7.03-25.74) | N/A | N/A |
| Day of Week | Monday^a^ | 10.33  (7.06-15.10) | N/A | N/A |
|  | Tuesday | 21.67  (14.69-31.97) | N/A | N/A |
|  | Wednesday | 16.98  (11.37-25.36) | N/A | N/A |
|  | Thursday | 13.40  (9.32-19.26) | N/A | N/A |
|  | Friday | 22.19  (15.17-32.46) | N/A | N/A |
|  | Saturday | 19.45  (14.60-25.92) | N/A | N/A |
|  | Sunday | 18.55  (13.70-25.11) | N/A | N/A |
| Hour | 06-11^a^ | 13.42  (10.32-17.46) | N/A | N/A |
|  | 12-17 | 18.78  (16.00-22.04) | N/A | N/A |
|  | 18-20 | 15.42  (9.40-25.30) | N/A | N/A |
|  | 21-5 | 301.40  (13.06-6954.88) | N/A | N/A |
| WBGT-Summer | WBGT≦18 | 52.09  (27.27-99.48) | 28<WBGT≦31 | 20<WBGT≦22 (0.500),  22<WBGT≦24 (0.336) |
|  | 18<WBGT≦20 | 19.55  (13.71-27.88) | N/A | N/A |
|  | 20<WBGT≦22 | 16.24  (13.72-19.24) | N/A | N/A |
|  | 22<WBGT≦24 | 14.68  (10.98-19.64) | N/A | N/A |
|  | 24<WBGT^a^ | 11.72  (3.11-44.17) | N/A | N/A |
| Month | April-May | 40.69  (21.56-76.79) | 28<WBGT≦31 | August (0.430) |
|  | June | 29.77  (18.55-47.77) | 28<WBGT≦31 | August (0.912) |
|  | July | 17.17  (14.13-20.86) | N/A | N/A |
|  | August^a^ | 13.05  (10.50-16.21) | N/A | N/A |
|  | September-October | 18.28  (10.51-31.79) | N/A | N/A |
| Region | Hokkaido | 79.00  (27.04-230.82) | 28<WBGT≦31 | N/A |
|  | Tohoku | 28.53  (17.74-45.89) | 28<WBGT≦31 | N/A |
|  | Kanto-Koshin | 16.71  (12.48-22.38) | N/A | N/A |
|  | Hokuriku | 38.61  (21.48-69.39) | 28<WBGT≦31 | N/A |
|  | Tokai | 18.27  (12.15-27.49) | N/A | N/A |
|  | Kinki | 15.43  (11.09-21.48) | N/A | N/A |
|  | Chugoku | 14.08  (8.49-23.36) | N/A | N/A |
|  | Shikoku | 14.03  (8.09-24.33) | N/A | N/A |
|  | Northern Kyushu | 12.85  (9.48-17.40) | N/A | N/A |
|  | Southern Kyushu and Amami^a^ | 11.90  (6.82-20.77) | N/A | N/A |
|  | Okinawa | 14.57  (3.38-62.75) | N/A | N/A |
| Location | Gymnasium/Indoor sports ground | 10.09  (7.52-13.55) | N/A | N/A |
|  | Out-of-school playgrounds and stadiums | 28.32  (22.04-36.39) | 28<WBGT≦31 | Gymnasium/Indoor sports ground (0.272),  Out-of-school sports halls (4.65e-2),  Other (1.08e-2) |
|  | Out-of-school sports halls | 7.78  (5.17-11.70) | N/A | N/A |
|  | Parks and amusement parks^a^ | 3.98  (0.34-47.15) | N/A | N/A |
|  | Playgrounds and schoolyards | 22.00  (17.62-27.47) | 28<WBGT≦31 | Gymnasium/Indoor sports ground (0.970),  Out-of-school sports halls (0.235),  Other (4.26e-2) |
|  | Roads | 33.64  (13.66-82.85) | 28<WBGT≦31 | Other (3.36e-2) |
|  | Other | 5.00  (2.57-9.72) | N/A | N/A |
| School | Junior high school | 17.48  (14.45-21.14) | N/A | N/A |
|  | High school^a^ | 16.80  (14.01-20.13) | N/A | N/A |
| Year | 2010^a^ | 5.87  (0.90-38.35) | N/A | N/A |
|  | 2011 | 13.08  (8.54-20.04) | N/A | N/A |
|  | 2012 | 13.49  (9.04-20.14) | N/A | N/A |
|  | 2013 | 16.30  (10.92-24.34) | N/A | N/A |
|  | 2014 | 14.32  (9.57-21.44) | N/A | N/A |
|  | 2015 | 20.18  (13.35-30.51) | N/A | N/A |
|  | 2016 | 21.19  (14.12-31.80) | N/A | N/A |
|  | 2017 | 9.10  (6.20-13.37) | N/A | N/A |
|  | 2018 | 18.54  (13.23-25.99) | N/A | N/A |
|  | 2019 | 27.97  (17.65-44.32) | 28<WBGT≦31 | 2017 (0.272) |

Abbreviations: Bonf., Bonferroni; CI, confidence interval; EV, explanatory variable; OR, odds ratio; WBGT, wet-bulb globe temperature (℃), α: significance level.

^a^ These EV2s were excluded from the comparison because their mean odds ratios were smaller than those of any of the EV2s for the same EV1.
